# Interferon-induced transmembrane protein 2 is a prognostic marker in colorectal cancer and promotes its progression by activating the PI3K/AKT pathway

**DOI:** 10.1007/s12672-024-01040-x

**Published:** 2024-05-27

**Authors:** Yonggang Liu, Jiyun Liang, Xi Li, Junyong Huang, Jiangyuan Huang, Jiale Wang

**Affiliations:** Department of Oncology, Shunde Hospital, Southern Medical University (The First People’s Hospital of Shunde), No.1 Jiazi Road, Shunde District, Foshan, 528308 Guangdong People’s Republic of China

**Keywords:** Interferon-induced transmembrane protein 2, Colorectal cancer, PI3K/AKT pathway, Proliferation, Metastasis

## Abstract

**Background:**

Interferon-induced transmembrane protein 2 (IFITM2) is involved in repressing viral infection. This study aim to investigate the expression of IFITM2 in colorectal cancer (CRC) and explore its effect on cell proliferation, migration, and invasion.

**Methods:**

We analyzed The Cancer Genome Atlas (TCGA) database for IFITM2 expression in colorectal cancer and used western blots to detect IFITM2 protein in specimens and cell lines of colorectal cancers. To assess the association between IFITM2 and clinical features, both univariate and multivariate cox regression analysis were conducted. Kaplan–Meier plots were used in the TCGA database to assess IFITM2 gene expression's prognostic significance. Silencing IFITM2 in SW480 and HCT116 cells was achieved by transient transfection with siRNA. Proliferation of CRCs was examined using Cell Counting Kit-8. The effect of IFITM2 on the migration and invasion of CRC cells was studied using wound healing and transwell assays. Gene set enrichment analysis (GSEA) was used to examine IFITM2-associated pathways and Western blotting was used to confirm it.

**Results:**

IFITM2 was over-expressed in the CRC tissues and cells, with high IFITM2 expression related to the tumor N, M, and pathologic stages. The presence of IFITM2 significantly impacted patient survival in CRC. The proliferation of SW480 and HCT116 cells was suppressed when IFITM2 was silenced, resulting in weakened migration and invasion of CRC cells. GSEA analysis showed that IFITM2 was positively related to the phosphoinositide 3-kinase (PI3K)/AKT pathway, and western blot results confirmed that IFITM2 activated it.

**Conclusions:**

IFITM2 was over-expressed in CRC and modulated the PI3K/AKT pathway to promote CRC cells proliferation and metastasis.

**Supplementary Information:**

The online version contains supplementary material available at 10.1007/s12672-024-01040-x.

## Introduction

Colorectal cancer (CRC) is a common malignant tumor of the gastrointestinal tract that seriously threatens global health. There is a high rate of mortality and morbidity associated with CRC in Chinam [1]. The main cause of death in patients with colorectal cancer is excessive proliferation and metastasis of tumor cells [2]. Therefore, novel biomarkers and targeted therapies should be explored for improvements in therapeutic efficacy.

Studies have confirmed that aberrantly activated signaling pathways play a key role in tumor development [3]. Phosphoinositide-3-kinase/Akt (PI3K/AKT) signaling pathway is one of the most classical pathways involved in tumorigenesis, which is widely involved in various biological functions, such as tumor metabolism, proliferation, motility, and survival [4, 5]. The study conducted by Danielsen et al. demonstrated that the activation of the PI3K/AKT signaling pathway facilitates cellular proliferation, invasion, and chemoresistance in CRC cells [6]. Thus, identifying potential regulatory genes for the PI3K/AKT signaling pathway and understanding its molecular mechanisms will provide a new direction for the treatment of CRC. Interferon-induced transmembrane proteins (IFITMs) are interferon-induced transmembrane proteins with molecular weights between 10 and 15kD. There are four functional members in the IFITM family: IFITM1, IFITM2, IFITM3, and IFITM5 [7, 8]. IFITMs were originally found to play a role in eukaryotic antiviral responses, and recent studies have confirmed that they play a role in tumor development. According to some studies, IFITM2 is abnormally expressed in esophageal cancer, lung cancer, gastric cancer, glioma, and affects biological functions [9–12]. Li et al. found that the KLF4 gene could negatively regulate IFITM3 to inhibiting colon cancer metastasis [13]. Andreu et al. showed that IFITM2 gene expression is significantly up-regulated specifically in colorectal tumors and thus may be a useful diagnostic tool in these tumors [14]. However, whether IFITM2 can induce tumour progression by PI3K/AKT signaling pathway in CRC remains to be elucidated.

The objective of our study was to assess the expression of IFITM2 in CRC cells and its impact on cell proliferation, migration, and invasion. Additionally, we aimed to confirm the role of IFITM2 in promoting malignant traits in CRC through the IFITM2-PI3K/AKT signaling pathway. The present study provides new insights to the discovery of prognostic biomarker and therapeutic target for CRC patients.

## Materials and methods

### The Cancer Genome Atlas (TCGA) datasets

Patients with CRC (n = 698) were retrieved via the R package (V3.6.2) from the TCGA database (www.tcga-data.nci.nih.gov/tcga/), including clinical and transcriptome sequencing data. Insufficient information regarding overall survival (OS), TNM stage, and gene expression data was excluded. Lastly, we screened 644 patients for cancer tissue and adjacent tissue in this study, including 50 pairs of cancer tissue and adjacent tissue.

### Bioinformatic analysis

Differential expression levels of IFITM2 in 36 cancer types were determined using the Tumor IMmune Estimation Resource (TIMER), a web resource for systematical evaluations of the clinical impact of different immune cells in diverse cancer types (https://cistrome.shinyapps.io/timer/) [15]. IFITM2 mRNA levels in the TCGA-colon adenocarcinoma rectum adenocarcinoma (COADREAD) datasets were analyzed via the The University of Alabama at Birmingham CANcer data analysis Portal (UALCAN) [16], and Gene Expression Profiling Interactive Analysis (GEPIA) database [17]. In order to analyze the IFITM2 protein levels, the Human Protein Atlas (https://www.proteinatlas.org/) was used. A Kaplan–Meier plot (https://kmplot.com/analysis/) was used to determine the effect of IFITM2 on survival rates [18]. TIMER 2.0 (https://cistrome.shinyapps.io/timer/) [19] was used to analyze the relationship between IFITM2 expression and tumor immune infiltration (TII). Gene set enrichment analysis (GSEA) [20] was conducted to identify the biological and functional pathways associated with high and low IFITM2 expression.

### Cell culture and treatment

The colorectal cancer cell lines HT29, SW480, HCT116, LoVo, RKO, and NCM460, were supplied by SIBS, CAS (Shanghai, China). All cells were grown in 6-well plates containing RPMI 1640 medium with 10% fetal bovine serum (FBS, HyClone, USA), and maintained at 37 °C and 5% CO_2_.

### Reverse transcription-quantitative real-time polymerase chain reaction (RT-qPCR) assays

Total RNA was extracted using TRIzol reagent (TakaraBio, Japan). cDNA synthesis was initiated with the First Strand cDNA Synthesis kit (TakaraBio) following the manufacturer’s instructions. RT-qPCR was conducted using a LightCycler 480 v1.5 real-time PCR system (Roche, Penzberg, Germany). The primers used were:

Human IFITM2 Forward: 5ʹ—TGTATCCCACGTACTCTATCTTCC—3ʹ,

Reverse: 5ʹ—GGACAGGGCGAGGAATGG—3ʹ;

Human GAPDH Forward: 5ʹ—ACCCAGAAGACTGTGGATGG-3ʹ,

Reverse: 5ʹ—TCTAGACGGCAGGTCAGGTC—3ʹ.

### Western blotting

Western blotting was performed according to the previous standard protocol. The primary antibodies used were: IFITM2 (1:500, A15133, Abclonal, Woburn, MA, USA), p-PI3K (1:1000, ab32089, Abcam, UK), p-AKT (1:1000, ab8933, Abcam, UK), PI3K (1:1000, ab140307, Abcam, UK), AKT (1:1000, ab38449, Abcam, UK) and GAPDH (1:2000, ab9485, Abcam, UK). The secondary antibody was an HRP-labeled anti-rabbit IgG antibody (1:5000, 7074P2, Cell Signaling Technology, USA). Femto enhanced chemiluminescence kit (#FD8030, FDbio, Shenzhen, China) was used to visualize the membranes. Finally, protein blots were analyzed using ImageJ software (NIH, MD, USA).

### Small interfering RNA (siRNA) transfection

SW480 and HCT116 cells were transfected with siRNAs (Sangon Biotech, Shanghai, China) using Lipofectamine 2000 (Invitrogen, Carlsbad, CA, USA). The siRNA sequences were as follows: siIFITM2, CCAUUCUGCUCAUCAUCAUTT. siIFITM2 efficiency was evaluated using real-time PCR and western blotting.

### Cell Counting Kit-8 (CCK-8) assays

Each experimental group's logarithmic growth phase cells were trypsinized, resuspended in complete medium, and cultured in 96-well plates. According to the manufacturer's instructions, Cell Counting Kit-8 reagent (Abcam) was used to assess cell proliferation. Microplate readers (Molecular Devices, Rockford, IL, USA) were used to detect optical density values at 490 nm.

### Migration assays

In accordance with the kit's instructions, migration assays were performed in a Transwell insert chamber (8-mm pore size; Corning, NY, USA). The upper chamber was pre-coated with Matrigel (Dow Corning) by 10%. The cells (5 × 104 cells/well) were transferred to the top chamber and incubated for 12 h at 37 °C. Inverted microscopes were used to capture images of invaded cells.

### Wound healing assays

The SW480 and HCT116 cell lines were grown overnight in 12-well plates (5 104 cells per well). When the cells reached 90% confluence, line-shaped wounds were created by scratching the monolayer with a sterile 10 µL pipette tip. Under an inverted microscope, the scratched wound areas were observed after incubation for 12 h in RPMI 1640 medium containing 1% FBS.

### Statistical analysis

All data were analyzed using R package v3.6.2 and SPSS version 20.0 (IBM, Chicago, IL, USA). The data are presented as mean ± SEM. Comparison between the two groups was performed by the Student’s t-test. The statistical significance was set at p < 0.05.

## Results

### Patient’s clinical features

The patient’s information (e.g., age, sex, TNM stage, histological grade, survival status, and survival time) was obtained from the TCGA-COADREAD database. After excluding cases with missing data, 644 patients were ultimately included (Table 1).

**Table 1:** Clinical Characteristics of Patients

Characteristic	Low expression of IFITM2	High expression of IFITM2
n	322	322
Age, n (%)		
< = 65	139 (21.6%)	137 (21.3%)
> 65	183 (28.4%)	185 (28.7%)
Gender, n (%)		
Female	156 (24.2%)	145 (22.5%)
Male	166 (25.8%)	177 (27.5%)
T stage, n (%)		
T1	5 (0.8%)	15 (2.3%)
T2	62 (9.7%)	49 (7.6%)
T3	215 (33.5%)	221 (34.5%)
T4	39 (6.1%)	35 (5.5%)
N stage, n (%)		
N0	179 (28%)	189 (29.5%)
N1	76 (11.9%)	77 (12%)
N2	65 (10.2%)	54 (8.4%)
M stage, n (%)		
M0	229 (40.6%)	246 (43.6%)
M1	43 (7.6%)	46 (8.2%)
Pathologic stage, n (%)		
Stage I	56 (9%)	55 (8.8%)
Stage II	112 (18%)	126 (20.2%)
Stage III	98 (15.7%)	86 (13.8%)
Stage IV	43 (6.9%)	47 (7.5%)
Age, median (IQR)	68 (57, 75)	68 (58.25, 77)

Over-expression of IFITM2 in CRC.

**Fig. 1:**
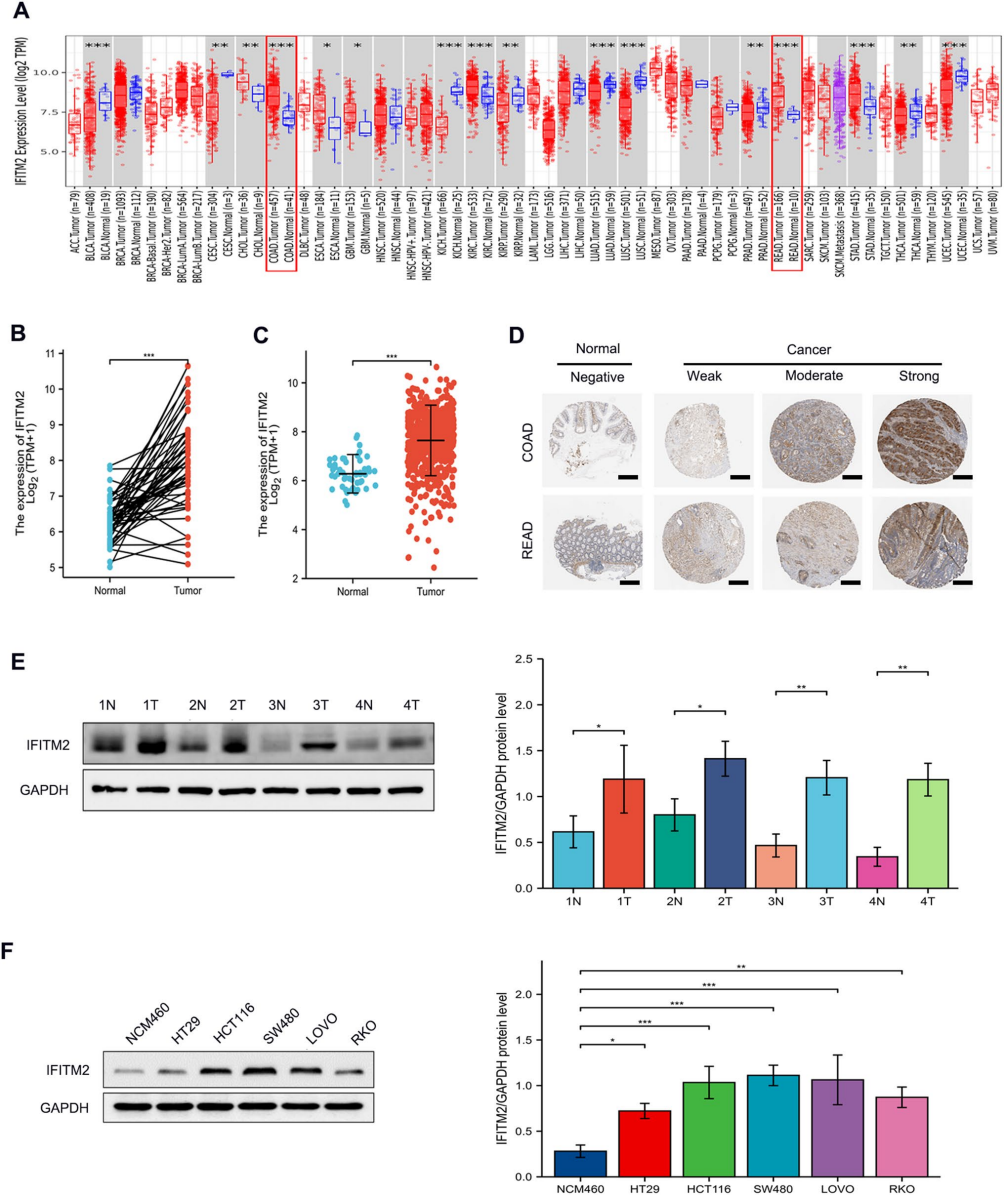
IFITM2 expression levels. **A** Differential expression of IFITM2 between tumor and normal tissue in 36 cancer types. **B** Compared with normal adjacent tissues, the expression level of IFITM2 mRNA in CRC paired and **C** unpaired. **D** Representative immunohistochemical staining pictures for IFITM2 with different staining intensities in CRC and normal tissues. **E**, **F** IFITM2 protein levels in CRC specimens and cells are upregulated. Scale bar: 200 μm. (*: p < 0.05, **: p < 0.01, and ***: p < 0.001)

**Fig. 2:**
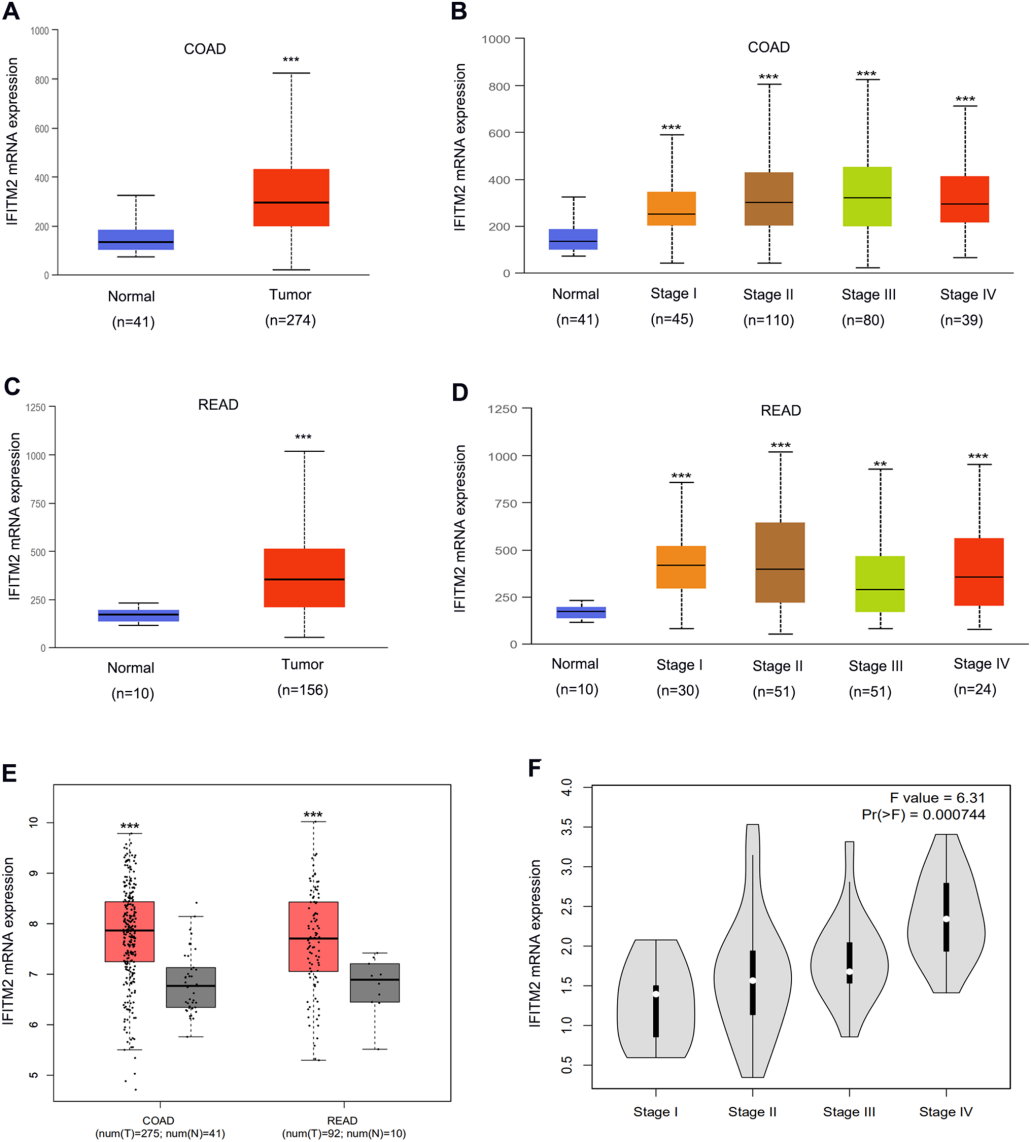
Analysis of IFITM2 mRNA expression using the UALCAN and GEPIA databases. **A** IFITM2 mRNA levels are upregulated in COAD samples from the UALCAN database. **B** Comparison of the IFITM2 mRNA expression levels in TNM in COAD. **C** IFITM2 mRNA levels are upregulated in READ samples from the UALCAN database. **D** Comparison of the IFITM2 mRNA expression levels in TNM stages in READ. **E** The IFITM2 mRNA expression levels are upregulated in CRC samples from the GEPIA database. **F** Comparison of the IFITM2 mRNA expression levels in the TNM stages in the GEPIA database. (**: p < 0.01, ***: p < 0.001)

According to TIMER analysis, IFITM2 was overexpressed in five types of cancer, including CRC (Fig. 1A). Furthermore, data from 50 CRC and adjacent healthy tissue pairs from the TCGA-COADREAD dataset (n = 644) showed that IFITM2 was over-expressed in CRC (p < 0.001; Fig. 1B). There were significant differences between the expression of IFITM2 in CRC and the adjacent healthy tissues (p < 0.001; Fig. 1C), implying that IFITM2 overexpression was specific to CRC. The Human Protein Atlas is used to determine the immunohistochemistry-based staining scores for normal and CRC tissues, according to three levels of tumor-specific staining: weak, moderate, and strong (Fig. 1D). CRC specimens and cells showed elevated levels of IFITM2 protein as assessed by Western blotting (Fig. 1E and F). The bioinformatics results were consistent with these findings. Additionally, we validated IFITM2 expression in the online tumor databases UALCAN and GEPIA, As shown in Fig. 2A-F, elevated mRNA expression of IFITM2 predicted advanced tumor stages.

### Relationship between IFITM2 expression and clinical features in CRC

The relationships between IFITM2 expression and clinical data in 644 CRC cases were analyzed. Logistic regression analysis was conducted after grouping based on the median expression of IFITM2. The findings demonstrated that high IFITM2 expression in CRC was markedly related to the tumor N (OR = 1.396 for N0 vs. N1 and N2), M (OR = 1.625 for M0 vs. M1), and pathologic stages (OR = 1.413 for stage I vs. stage II, stage III, and stage IV) (all p < 0.05; Table 2). This indicated that CRC patients with IFITM2 upregulation were more susceptible to advanced CRC than those without upregulation.

**Table 2:** Relation between IFITM2 expression and clinical characteristics

Characteristics	Total(N)	Odds Ratio(OR)	P value
Age (< = 65 vs. > 65)	644	1.135 (0.831–1.552)	0.426
Gender (Female vs. Male)	644	0.940 (0.689–1.281)	0.693
T stage (T1 vs. &T2T3&T4)	641	1.231 (0.838–1.812)	0.291
N stage (N0 vs. N1&N2)	640	1.396 (1.019–1.913)	0.038
M stage (M0 vs. M1)	564	1.652 (1.107–2.477)	0.014
Pathologic stage (Stage I vs. Stage II& Stage III&Stage IV)	623	1.413 (1.029–1.942)	0.033

### Univariate and multivariate Cox regression analyses of IFITM2 expression in CRC

**Fig. 3:**
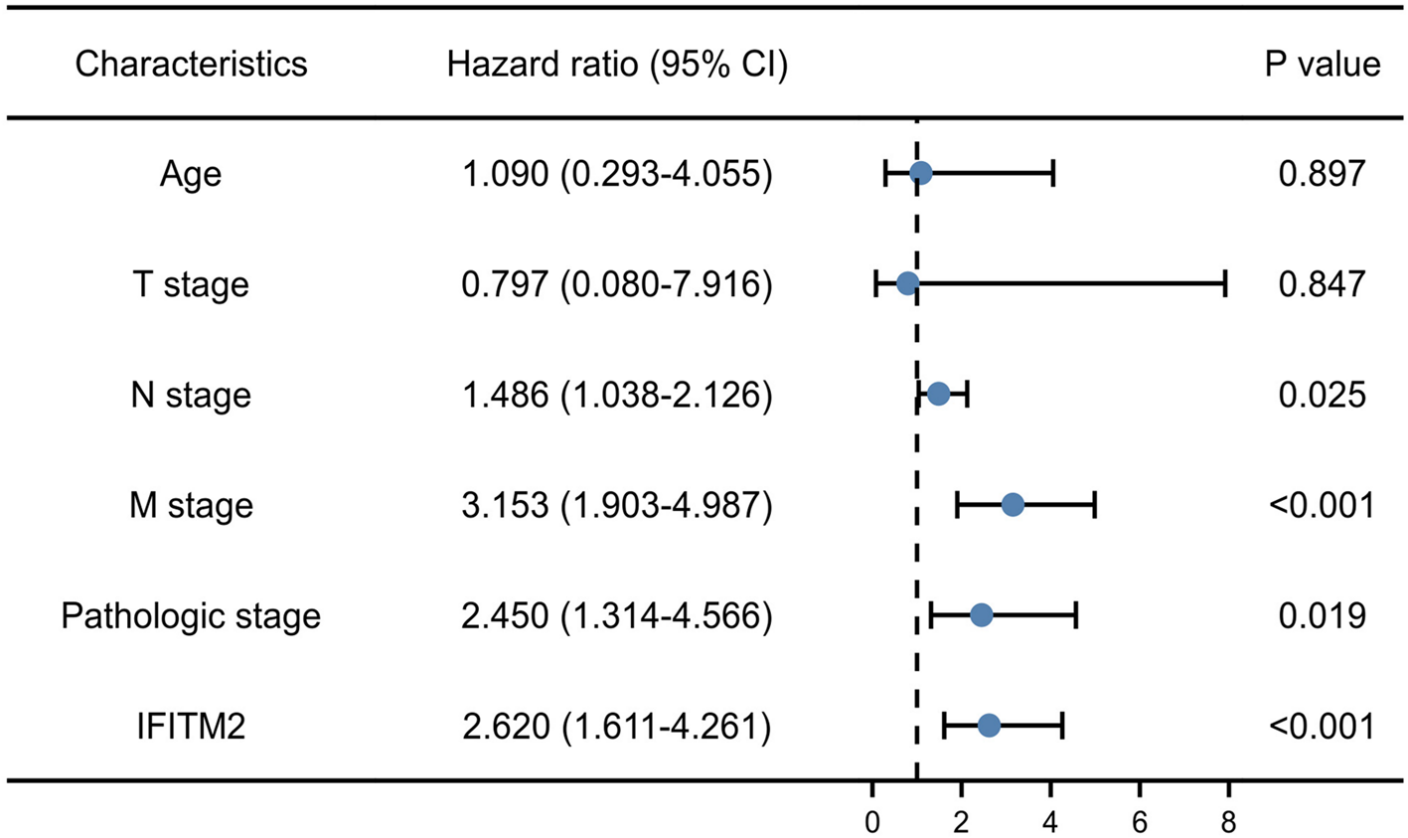
Forest plot of the correlation of IFITM2 expression with overall survival among patients with CRC

Univariate regression analysis revealed that the factors affecting OS in patients with gastric cancer were age (p < 0.001), T stage (p = 0.004), N stage (p < 0.001), M stage (p < 0.001), histologic grade (p < 0.001) and IFITM2 expression level (p < 0.001; Table 3). Multivariate regression analysis of these factors showed that N stage (HR = 1.486, p = 0.025), M stage (HR = 3.153, p < 0.001), pathologic stage (HR = 2.450, p = 0.019), and IFITM2 expression level (HR = 2.620, p < 0.001) were significant risk factors affecting survival in patients with CRC (Table 3 and Fig. 3).

**Table 3:** Univariate and multivariate Cox regression analysis for IFITM2 expression

Characteristics	Univariate analysis		Multivariate analysis	
	Hazard ratio (95% CI)	P value	Hazard ratio (95% CI)	P value
Age	1.939 (1.320–2.849)	< 0.001	1.090 (0.293–4.055)	0.897
Gender	1.054 (0.744–1.491)	0.769		
T stage	2.468 (1.327–4.589)	0.004	0.797 (0.080–7.916)	0.847
N stage	2.627 (1.831–3.769)	< 0.001	1.486 (1.038–2.126)	0.025
M stage	3.989 (2.684–5.929)	< 0.001	3.153 (1.903–4.987)	< 0.001
Pathologic stage	2.988 (2.042–4.372)	< 0.001	2.450 (1.314–4.566)	0.019
IFITM2	4.609 (2.804–7.577)	< 0.001	2.620 (1.611–4.261)	< 0.001

### Prognostic value of IFITM2 in CRC

**Fig. 4:**
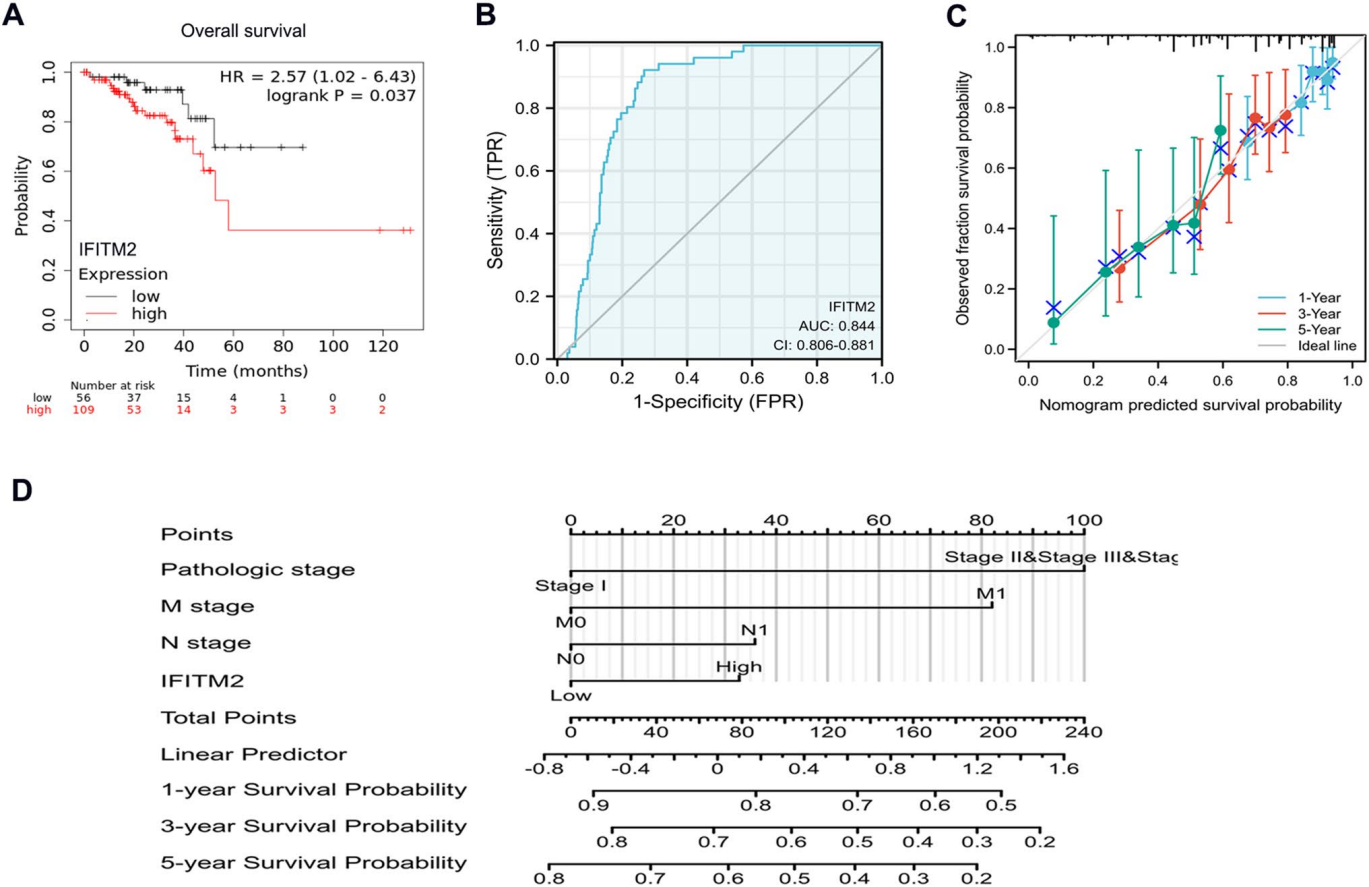
Prognostic value of IFITM2 in CRC. **A** Effect of IFITM2 expression on overall survival in patients with CRC based on TCGA-COADREAD database. **B** ROC analysis of the predictive effect of IFITM2 on CRC. **C** The calibration curve shows the predictive performance of the model constructed using multifactor Cox regression analysis. **D** Multivariate analysis nomogram based on the clinical characteristics of IFITM2 expression.

The Kaplan–Meier plotter was used to assess the effect of IFITM2 expression on survival time in CRC. From the TCGA-COADREAD database, IFITM2 downregulation was related to improved OS (HR = 2.57, p = 0.037, Fig. 4A). The diagnostic roles of IFITM2 in CRC were evaluated using receiver operating characteristic (ROC) curve analysis in the TCGA CRC and normal gastric specimens. The area under the ROC curve (AUC) analysis demonstrated that IFITM2 was specific and sensitive for the diagnosis of CRC (AUC = 0.844, CI  0.806–0.881, Fig. 4B). Concurrently, The calibration curve of the prediction model showed that the established lines of 1-, 3- and 5 year survival matched the ideal line at a high degree (Fig. 4C). The tumor’s pathologic stage, M stage, N stage, and IFITM2 expression levels were used to construct a clinical prognostic risk score for CRC. we constructed a prognostic nomogram to predict individual survival probability (Fig. 4D). All of these results indicated that IFITM2 expression level correlated with CRC prognosis.

### Correlation between IFITM2 expression levels and TII in CRC

**Fig. 5:**
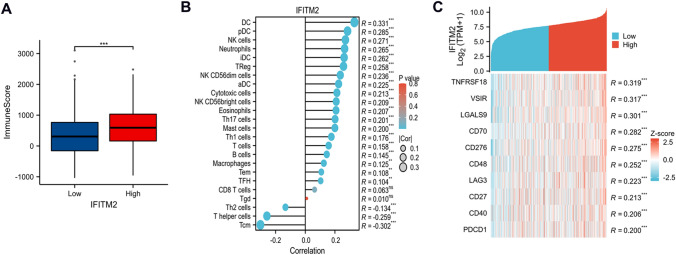
Relationship between IFITM2 expression and tumor immune infiltration in CRC. **A** Correlation between IFITM2 expression and immune score. **B** Comparison of immune infiltration levels of immune cells (including DCs, pDCs, NK cells and neutrophils) between the high- and low-IFITM2 expression groups. **C** Correlation between IFITM2 expression and clinical common immune checkpoint genes. (ns: No Significant, **: P < 0.01, ***: P < 0.001)

To assess TII in CRC patients with differential IFITM2 expression levels in the TCGA-COADREAD database, we used the TIMER database. Results showed a significant positive correlation between IFITM2 expression levels and immune scores (Fig. 5A). Additionally, IFITM2 expression was positively related to the infiltration level of dendritic cells (DCs) (R = 0.331), plasmacytoid dendritic cells (pDCs) (R = 0.285), NK cells (R = 0.271) and neutrophils (R = 0.265) (all p < 0.001; Fig. 5B). Notably, the following clinical common immune checkpoint genes were positively related to IFITM2 expression (all R > 0, p < 0.001): TNFRSF18, VSIR, LGALS9, CD70 and CD276 (B7-H3) (Fig. 5C). The results indicated that patients with IFITM2 upregulation may have a favorable response to treatment related to immune checkpoint blockade.

IFITM2-related signaling pathways based on Gene Set Enrichment analysis.

**Fig. 6:**
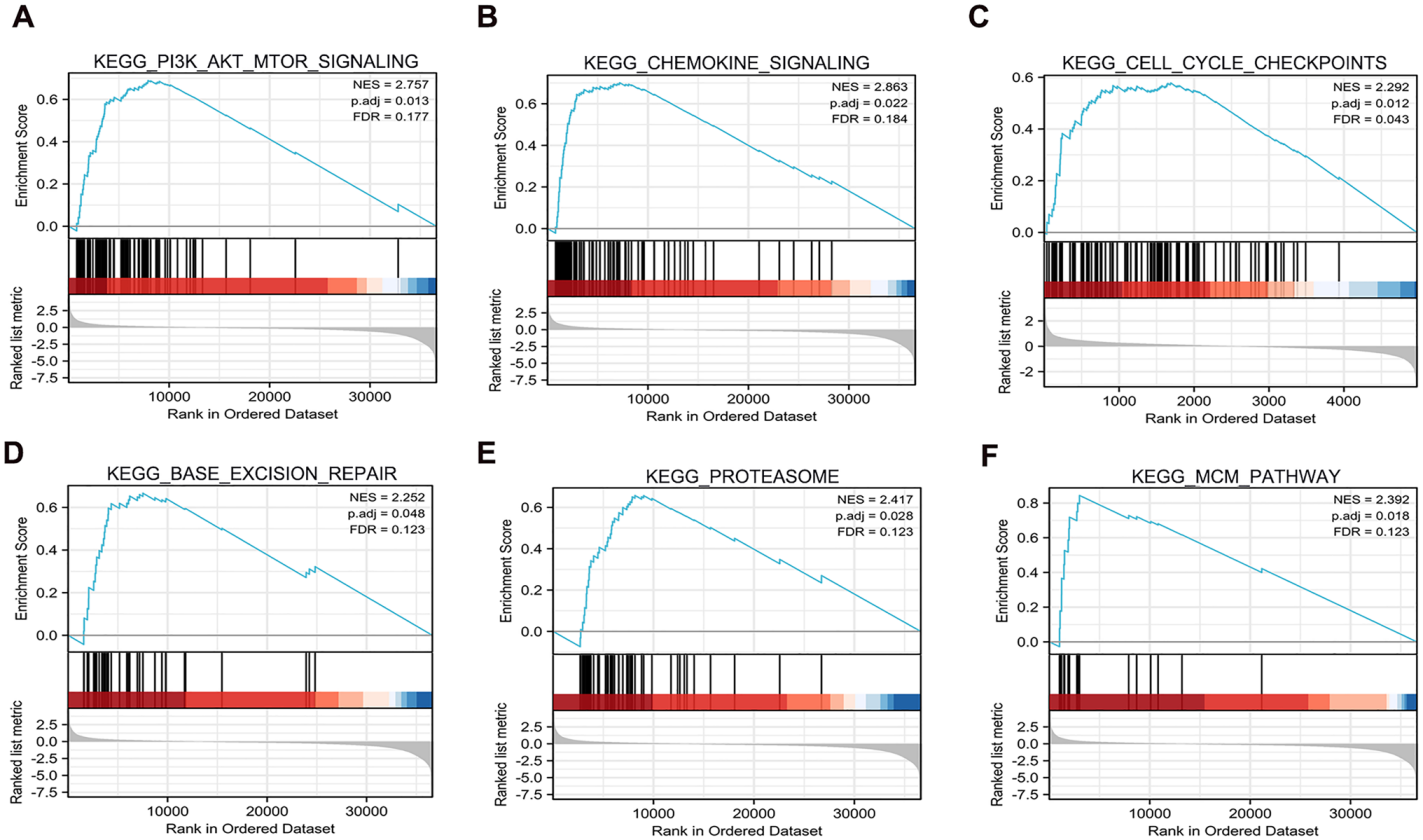
Functional pathway of GSEA enrichment analysis with high expression of IFITM2. **A** PI3K_AKT_MTOR_signaling; **B** Chemokine_signaling; **C** Cell_cycle_checkpoints; **D** Base_excision_repair; **E** Proteasome; **F**: MCM pathway

GSEA was used to determine KEGG pathway enrichment in groups with low-expression and high-expression of IFITM2. Gene sets with FDR q < 0.05 and NOM p < 0.05 were deemed significant. KEGG analysis indicated that IFITM2 was over-expressed in the PI3K_AKT_MTOR_signaling, Chemokine_signaling, Cell_cycle_checkpoints, Base_excision_repair, Proteasome and Mini-chromosome maintenance (MCM) pathway (Fig. 6A-F).

### IFITM2 knockdown inhibited the proliferation and migration of CRC cell lines

**Fig. 7:**
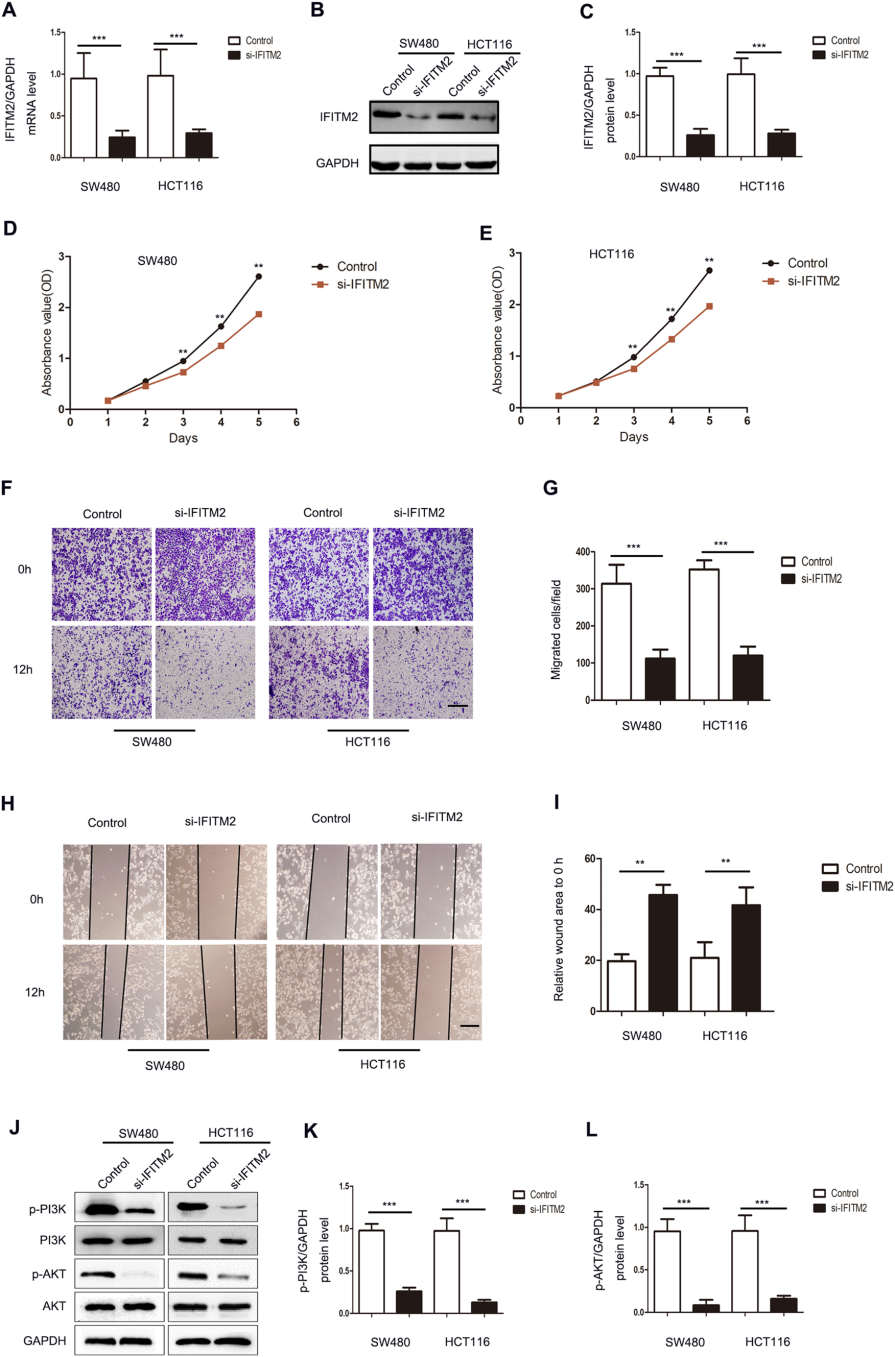
IFITM2-knockdown inhibits the proliferation and migration of CRC cell lines. **A**–**C** si-IFITM2 efficiency was measured using RT-qPCR and western blot. **D**–**E** Effect of IFITM2 knockdown on cell proliferation in vitro. **F**–**I** Migration assay and wound healing assay within 12 h of incubation show inhibited migration and scratch repair of IFITM2-silenced CRC cells. **J**–**L** Effect of IFITM2 on the PI3K/AKT pathway in CRC. Scale bar: 200 μm. (**: p < 0.01, ***: p < 0.001)

RT-qPCR and western blotting were used to determine the efficiency of si-IFITM2 in SW480 and HCT116 cell lines (Fig. 7A-C). The CCK-8 results showed reduced proliferation of si-IFITM2 cells than that of the control cells (p < 0.001, Fig. 7D and E). The effect of IFITM2 on the invasion and migration of gastric cancer was further investigated. Migration and wound healing assays within 12 h of incubation demonstrated attenuated invasion, migration, and scratch repair of IFITM2-knockdown CRC cell lines (Fig. 7E-I). A pathway enrichment analysis of GSEA revealed a positive association between IFITM2 and PI3K/AKT (p < 0.05, Fig. 6A); The expression of p-PI3K and p-AKT decreased after IFITM2 knockdown (p < 0.001, Fig. 7J-L), suggesting that IFITM2 knockdown inhibited the activation of PI3K/AKT pathway.

## Discussion

In the world, CRC is a malignant gastrointestinal tumor with high morbidity and mortality rates. Despite improvements in recent years, the 5-year survival rate for advanced cancer patients still falls below 10% [21]. Tumor proliferation and metastasis are important prognostic factors [22]. Consequently, finding key molecules that control tumor proliferation and metastasis could provide a new treatment strategy for improving the prognosis of colorectal cancer patients.

The IFITM2 belongs to the interferon-induced transmembrane protein family, and early studies focused on how the protein regulates antiviral responses [23]. In recent years, however, a growing number of studies have shown that IFITM2 is associated with tumor development and often plays an important role in oncogene expression. In renal clear cell carcinomas, Yang et al. observed high expression of IFITM2 resulting in tumor invasion and metastasis through lymph angiogenesis [24]. Our previous study showed that IFITM2 activates the TGF-β1 pathway in gastric cancer to promote epithelial-mesenchymal transition [25]. In CRC, however, expression and mechanisms of IFITM2 remain unclear. In this study, we confirmed that IFITM2 expression was present in 36 different types of cancer using data from the TCGA database. According to the pan-cancer analysis, IFITM2 expression varied between cancer types: the expression was significantly higher in colorectal cancer, gastric cancer, renal clear cell carcinoma, and bile duct carcinoma, but low expression in bladder urothelial carcinoma, renal chromophobe cell carcinoma, lung squamous cancer, lung adenocarcinoma, and endometrial cancer. Therefore, different tumors may exhibit different functional characteristics for IFITM2.

In the current study, we demonstrated overexpression of the IFITM2 gene in CRC compared with adjacent healthy tissue, which was associated with poor OS. IFITM2 expression in human CRC cells and clinical specimens was validated by Western blotting. These data were consistent with the bioinformatics data. In our analysis of 644 cases of CRC, higher IFITM2 expression was positively correlated with tumor N, M, and pathological stages. CRC patients with high IFITM2 expression had an increased risk in the late N, M, and pathological stages of the disease, suggesting that IFITM2 is a tumor-related gene. According to the ROC analysis, IFITM2 expression was a significant prognostic biomarker for CRC, while univariate and multivariate regression analyses further confirmed this finding. Taken together, IFITM2 may be used as a biomarker for oncogenes and prognosis.

It has become increasingly clear that immunotherapy is a promising treatment option for advanced colorectal cancer. According to recent research, immune infiltration levels affect immunotherapy efficacy [26–28]. Despite this, we know little about the correlation between IFITM2 and immune infiltrates. In our study, Data from 644 patients with CRC were downloaded from the TCGA database to analyze whether IFITM2 expression is related to the immune microenvironment of CRC. According to our analysis, IFITM2 expression is related to different immune marker sets and immune infiltration levels in CRC. The TIMER 2.0 database was analyzed to uncover a possible correlation between high expression of IFITM2 and immune cell infiltration. As our results showed, IFITM2 was positively correlated with the immune score. Additionally, IFITM2 expression was positively related to the infiltration level of DCs, pDCs, NK cells and neutrophils. Notably, the following clinical common immune checkpoint genes were positively related to IFITM2 expression. This study indicates that IFITM2 contributes to immune microenvironments of CRC and immunotherapy resistance.

IFITM2 is a potential biomarker in some cancers, but its functional mechanism is still elusive [24, 25, 29]. In this study, we demonstrated that interfering with IFITM2 expression inhibited the proliferative potential of colorectal cancer cells. Meanwhile, the migration and wound healing assays within 12 h of incubation demonstrated attenuated migration and scratch repair of IFITM2-knockdown CRC cells. These findings indicate that IFITM2 contributes to CRC proliferation, invasion, and metastasis.

Signaling pathways play a crucial role in tumor occurrence and development. According to our previous study, IFITM2 induces epithelial-mesenchymal transition by activating IGF1/IGF1R/STAT3 and TGF-β1/Smad2 pathways [25, 29]. Nonetheless, whether IFITM2 influences CRC development through inflammation has not been investigated. In order to investigate the molecular mechanism by which IFITM2 promotes CRC proliferation and metastasis, GSEA was conducted on IFITM2-associated signaling pathways in CRC. The results suggested that IFITM2 was possibly associated with the PI3K/AKT signaling pathway, Chemokine signaling, Cell cycle chenckpoints, Base excision repair, Proteasome, MCM pathway. These signaling pathways play important roles in the development and progression of CRC [30]. There are many important roles played by the PI3K/AKT signaling pathway in tumor metabolism, proliferation, epithelial-to-mesenchymal transition, and drug resistance. The classical PI3K/AKT pathway activates PI3K kinase by stimulation of growth factors, activation of oncogenes, or kinase stimulation. PI3K kinase activation further phosphorylates Akt, which promotes protein transcription by activating the mTORC1 complex [31–33]. As shown in this study, inhibition of IFITM2 significantly inhibited PI3K/AKT activation in CRC cells, suggesting that the cancer-promoting effect of IFITM2 in CRC may be related to its regulation of the PI3K/AKT signaling pathway.

Although this work provides a deeper understanding of the relationships between IFITM2 expression levels and prognosis for patients with CRC, this work has some important limitations. First, since this study only used an online dataset, the results may be influenced by selection bias. Second, as a large proportion of the data utilized in this analysis came from online sources, certain crucial clinical data of the patients was not obtained. Moreover, further research and thorough experimental validations are needed for all of the in vitro and in vivo experiments of this study. The biological functions and underlying mechanisms of how IFITM2 expression levels impact progression and patient prognosis in patients with CRC remain unclear and require additional study.

In conclusion, this study confirmed that IFIMT2 is highly expressed in CRC and the high expression of IFITM2 activates PI3K/AKT signaling pathway within tumor cells and promotes the proliferation and metastasis of CRC. Therefore, IFITM2 is expected to be an effective target to improve the prognosis of CRC patients, and this study also provides some experimental basis for the targeted intervention of PI3K/AKT pathway-related molecules in the treatment of tumor metastasis.

### Supplementary Information

Supplementary material 1.

## Data Availability

The datasets supporting the conclusions of this article are included within the article.
