# Supplementary figures and images for: Interferon-induced transmembrane protein 2 is a prognostic marker in colorectal cancer and promotes its progression by activating the PI3K/AKT pathway
Source: Discov Oncol. 2024 May 27;15:191. doi: 10.1007/s12672-024-01040-x (PMC11130111; doi:10.1007/s12672-024-01040-x)

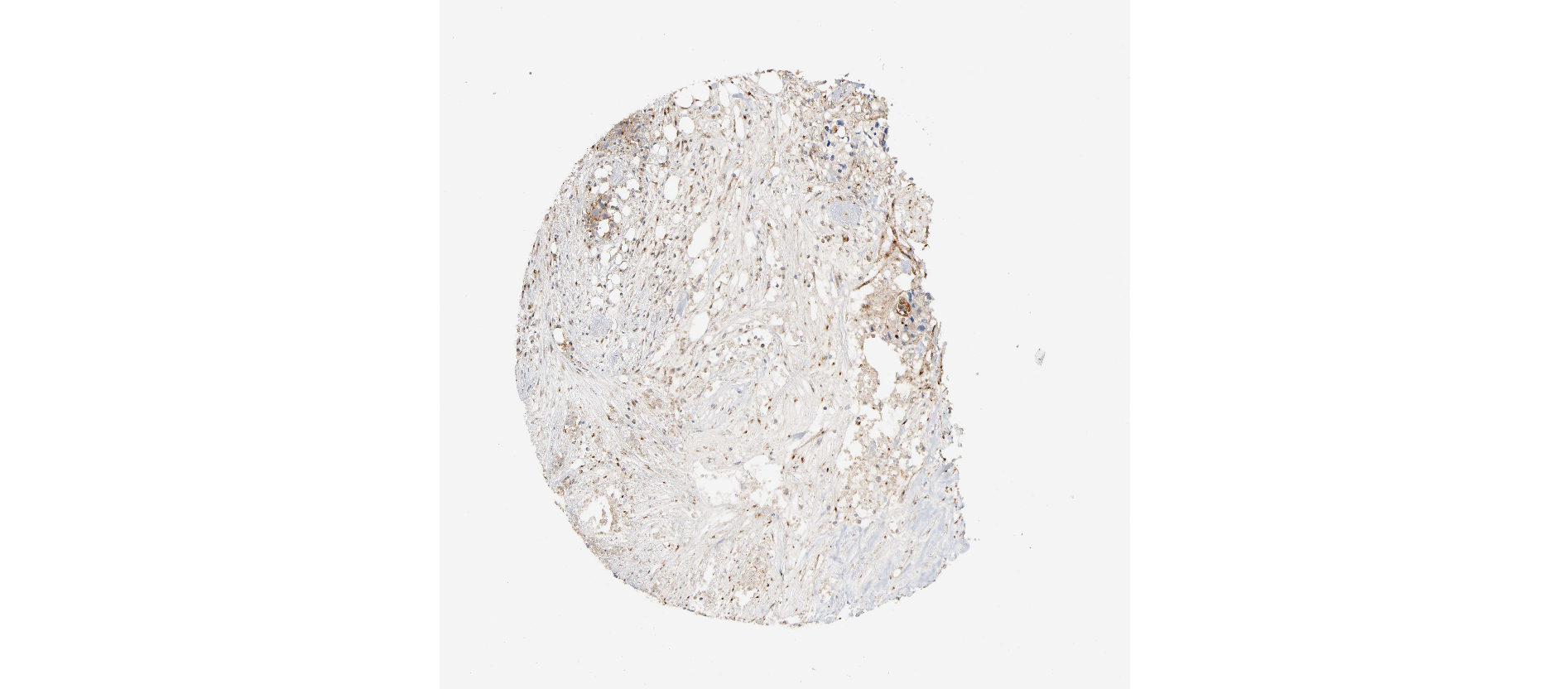

Supplement: Supplementary file 1 — Supplementary material 1. [file 12672_2024_1040_MOESM1_ESM.zip › original images/IHC/coad-m1.png]

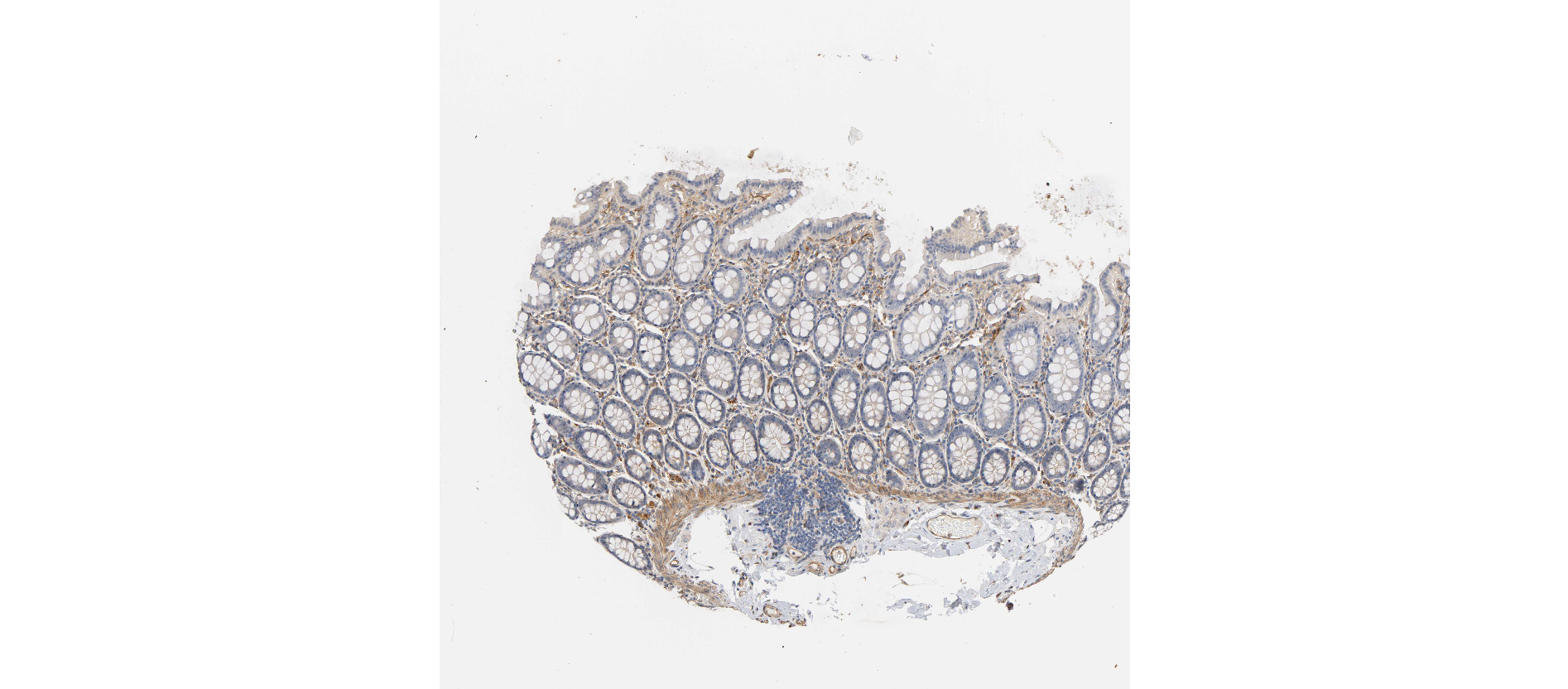

Supplement: Supplementary file 1 — Supplementary material 1. [file 12672_2024_1040_MOESM1_ESM.zip › original images/IHC/Colon nor-1.png]

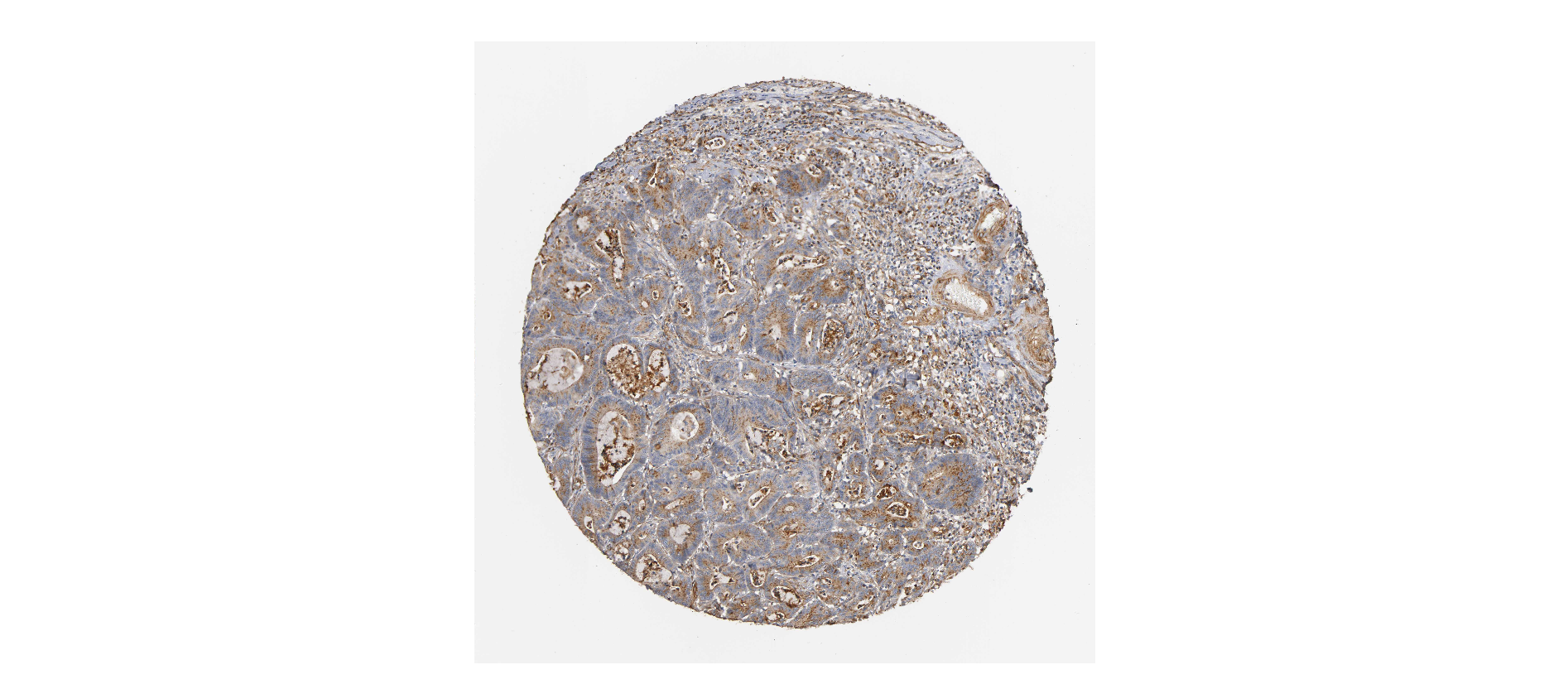

Supplement: Supplementary file 1 — Supplementary material 1. [file 12672_2024_1040_MOESM1_ESM.zip › original images/IHC/m1.png]

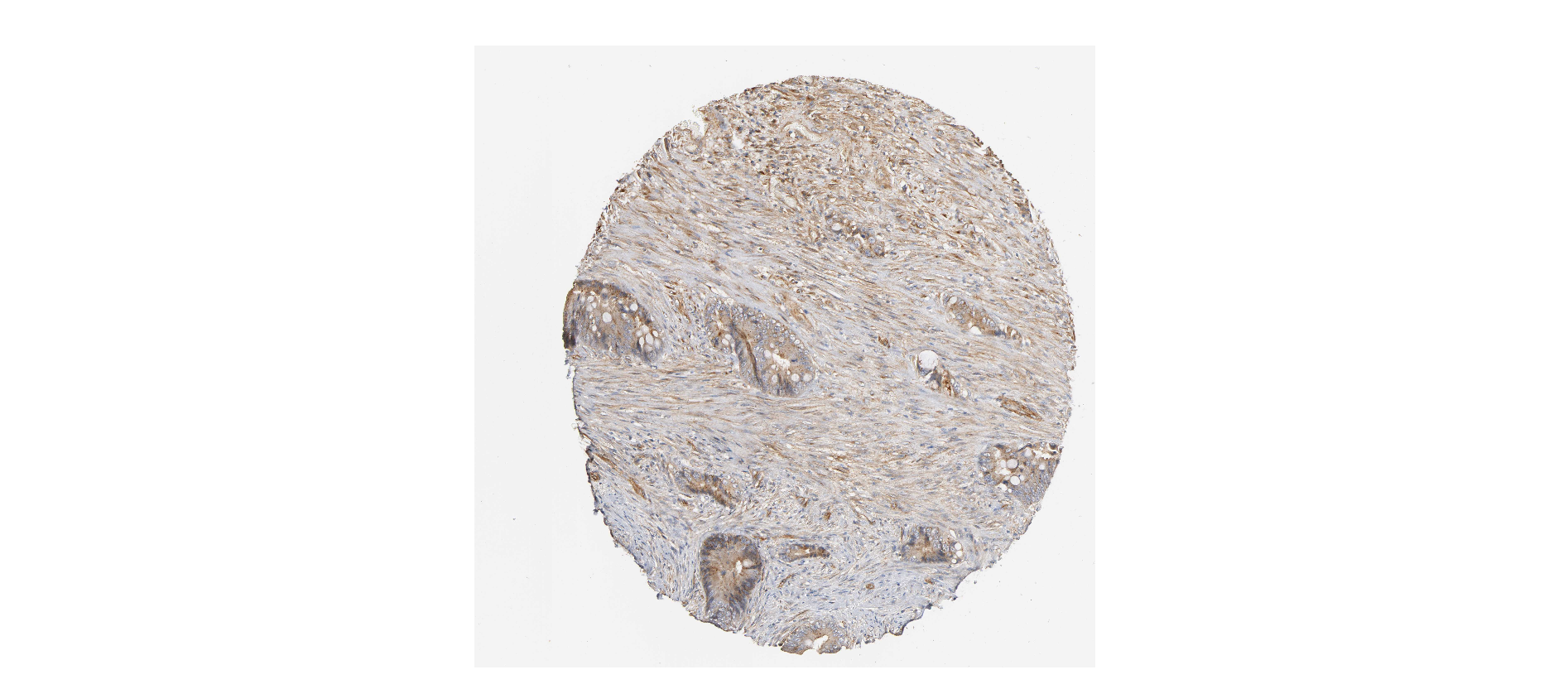

Supplement: Supplementary file 1 — Supplementary material 1. [file 12672_2024_1040_MOESM1_ESM.zip › original images/IHC/READ-M1.png]

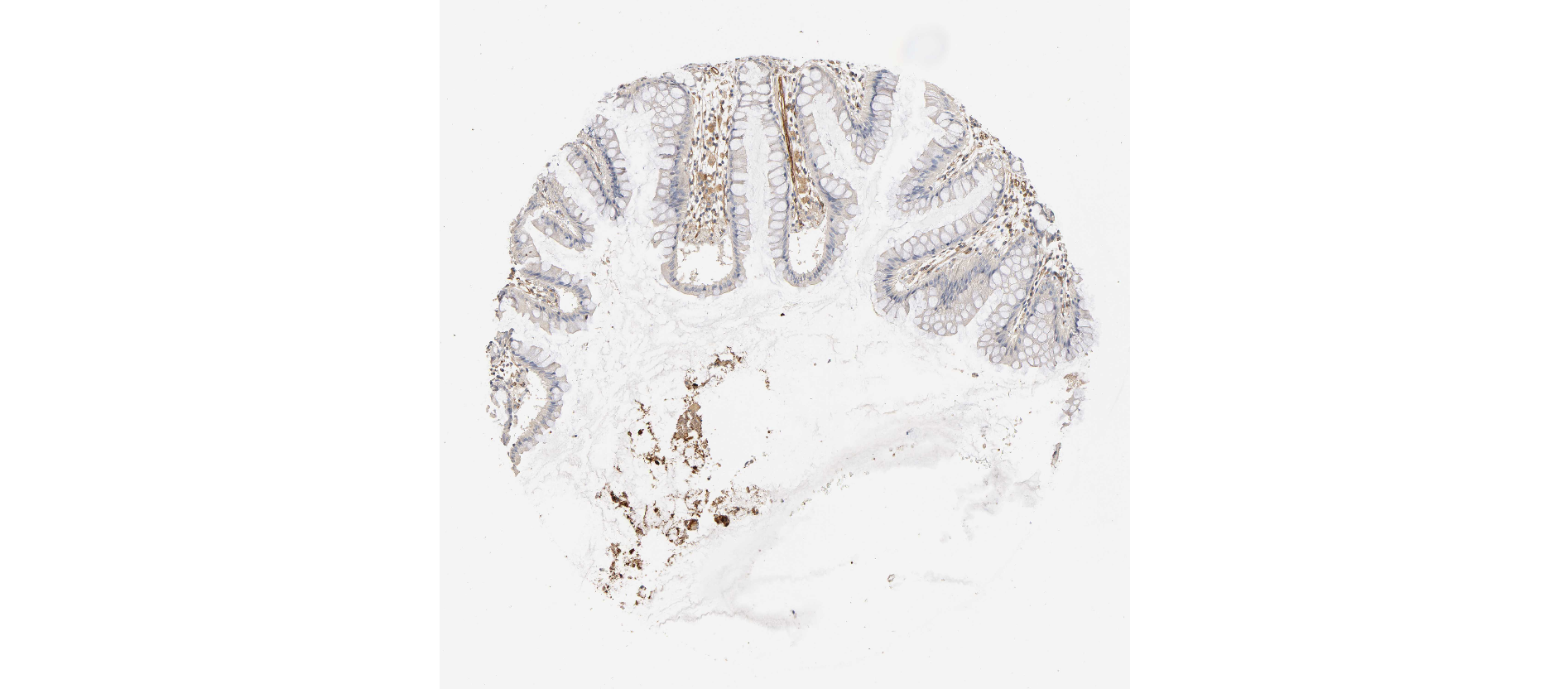

Supplement: Supplementary file 1 — Supplementary material 1. [file 12672_2024_1040_MOESM1_ESM.zip › original images/IHC/Rectum nor-1.png]

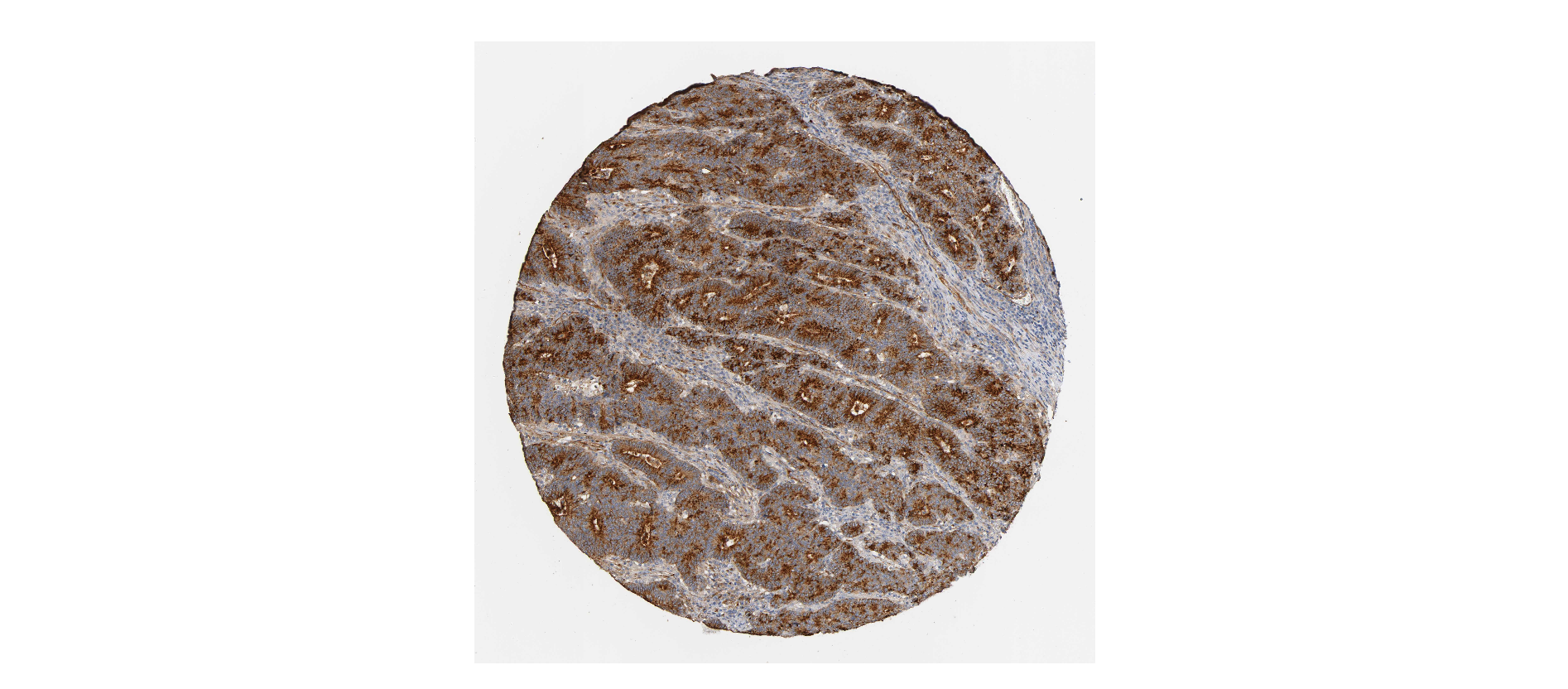

Supplement: Supplementary file 1 — Supplementary material 1. [file 12672_2024_1040_MOESM1_ESM.zip › original images/IHC/s1.png]

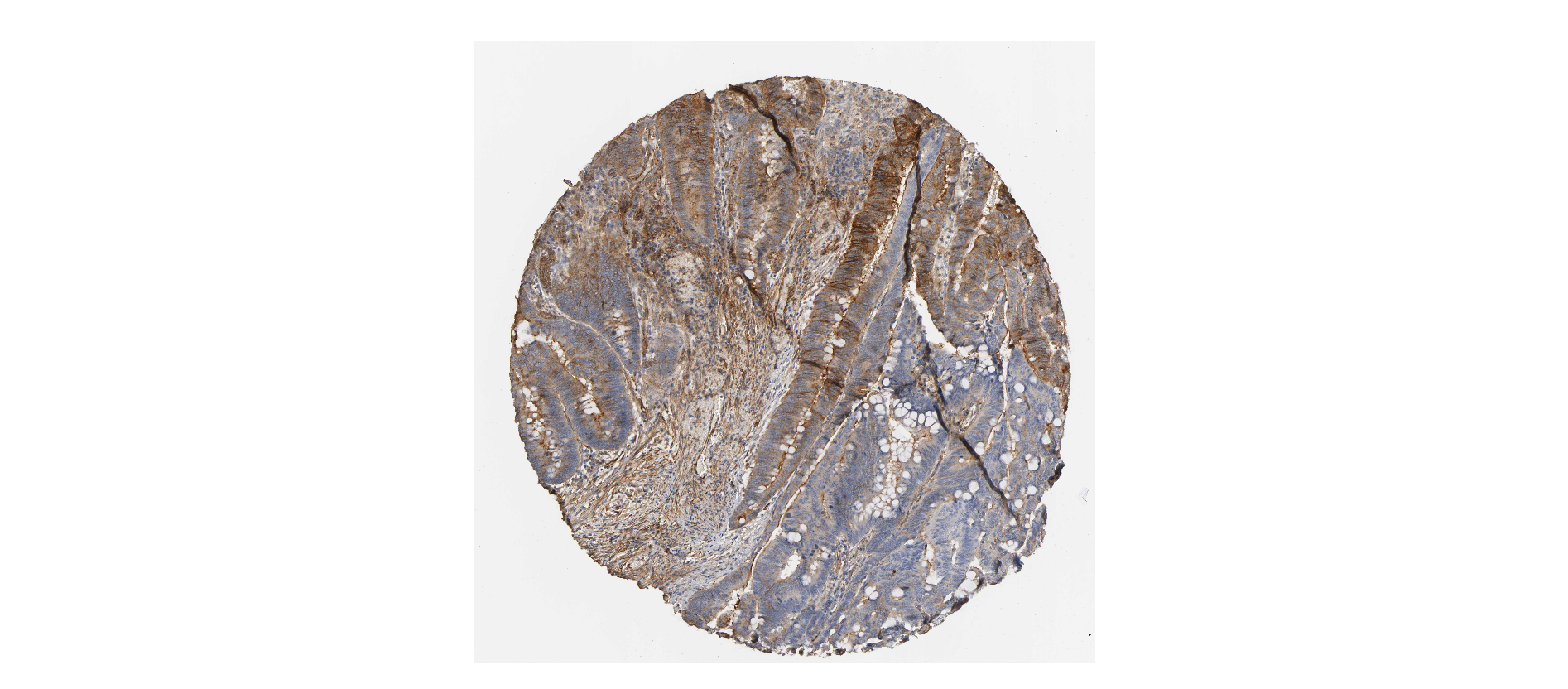

Supplement: Supplementary file 1 — Supplementary material 1. [file 12672_2024_1040_MOESM1_ESM.zip › original images/IHC/s2.png]

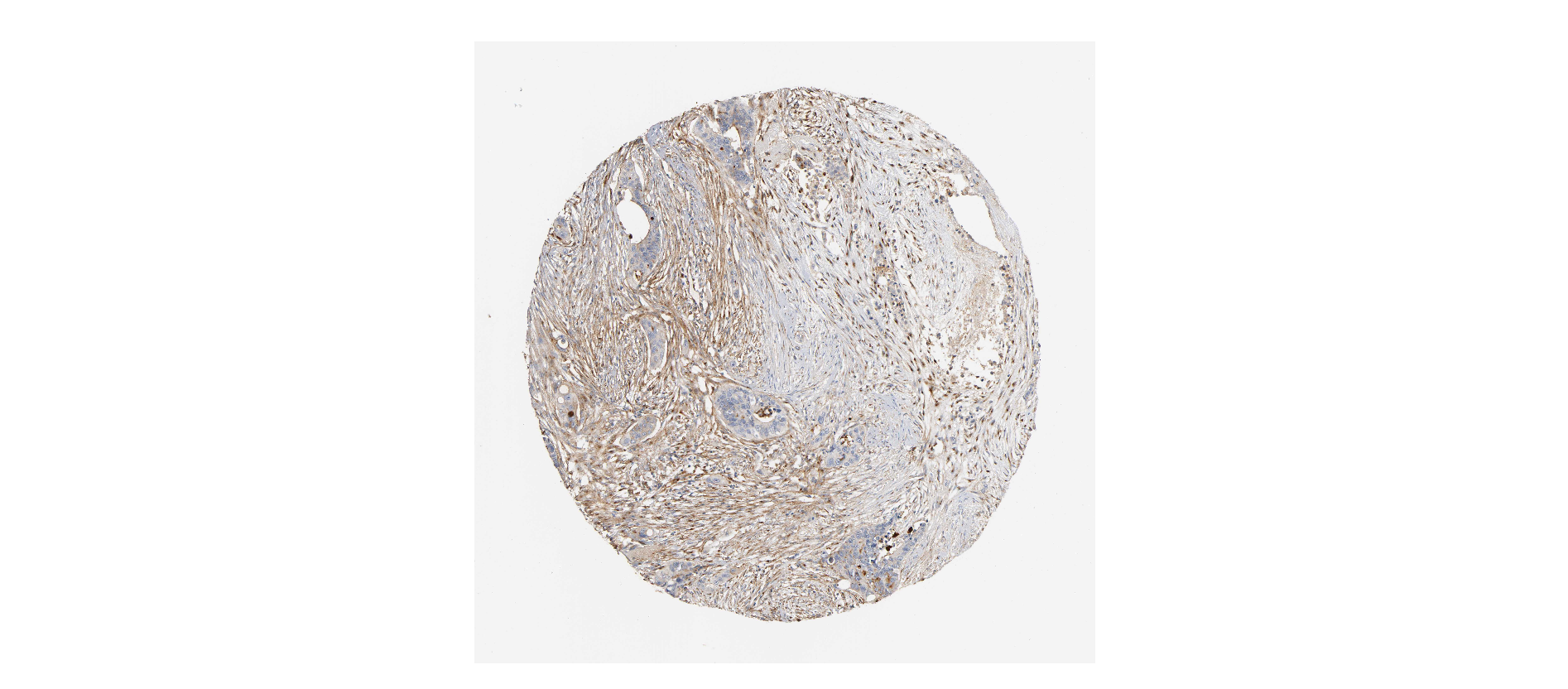

Supplement: Supplementary file 1 — Supplementary material 1. [file 12672_2024_1040_MOESM1_ESM.zip › original images/IHC/w2.png]

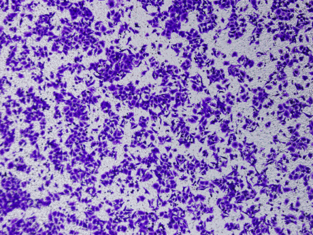

Supplement: Supplementary file 1 — Supplementary material 1. [file 12672_2024_1040_MOESM1_ESM.zip › original images/Migration assay/hct116-0h-siIFITM2.tif]

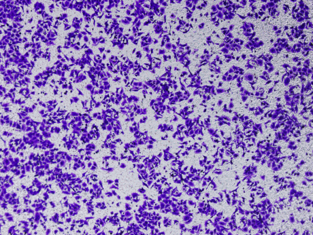

Supplement: Supplementary file 1 — Supplementary material 1. [file 12672_2024_1040_MOESM1_ESM.zip › original images/Migration assay/hct116-0h.tif]

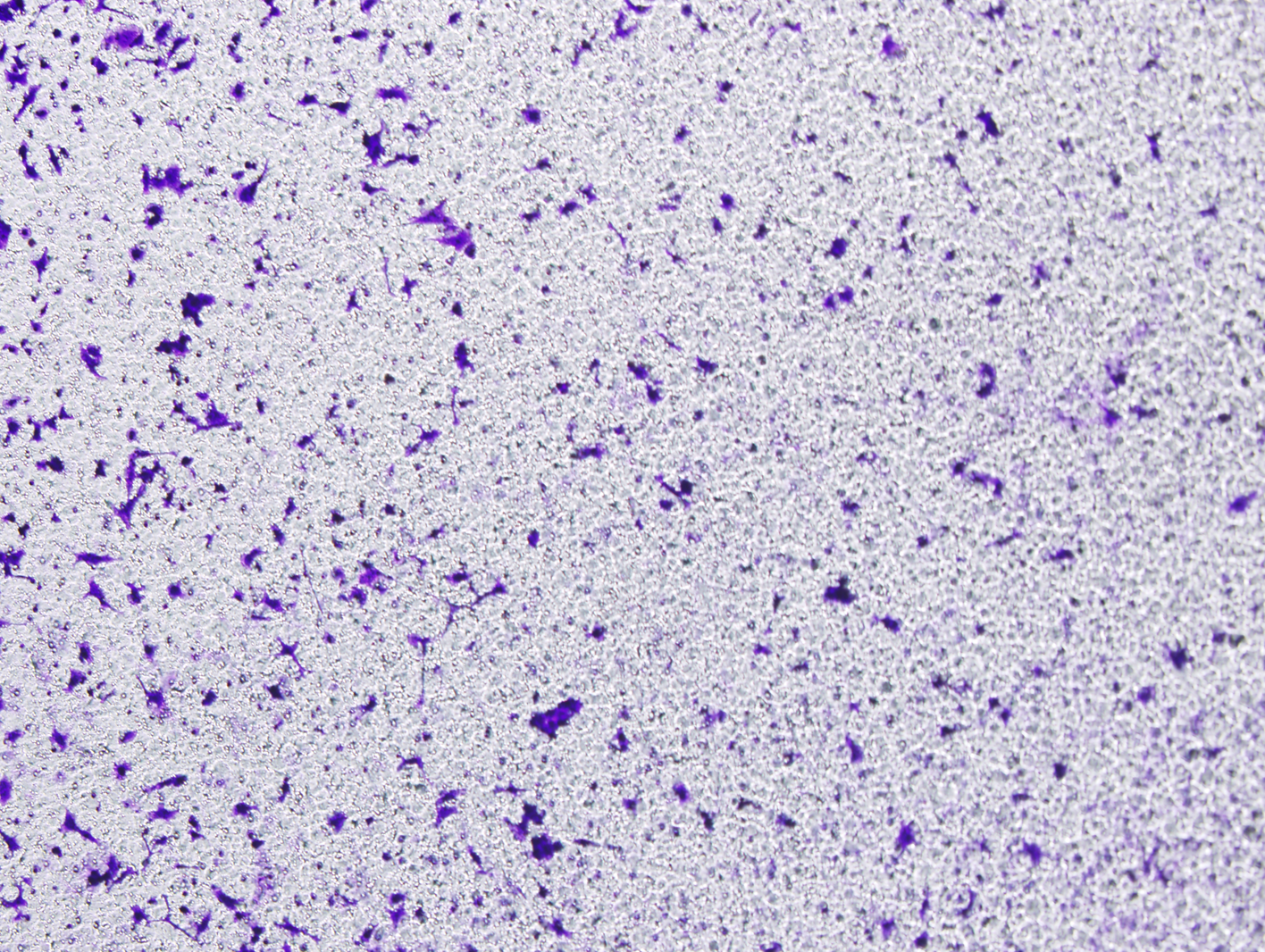

Supplement: Supplementary file 1 — Supplementary material 1. [file 12672_2024_1040_MOESM1_ESM.zip › original images/Migration assay/hct116-12h-siIFITM2.tif]

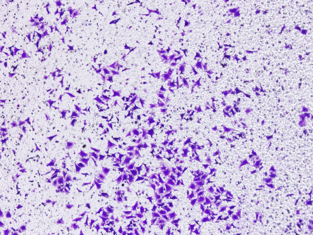

Supplement: Supplementary file 1 — Supplementary material 1. [file 12672_2024_1040_MOESM1_ESM.zip › original images/Migration assay/hct116-12h.tif]

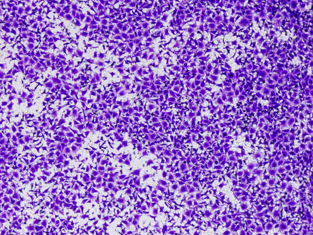

Supplement: Supplementary file 1 — Supplementary material 1. [file 12672_2024_1040_MOESM1_ESM.zip › original images/Migration assay/sw480-0h-siIFITM2.tif]

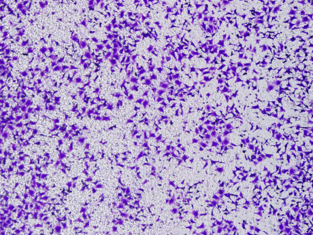

Supplement: Supplementary file 1 — Supplementary material 1. [file 12672_2024_1040_MOESM1_ESM.zip › original images/Migration assay/sw480-0h.tif]

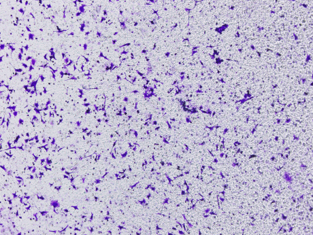

Supplement: Supplementary file 1 — Supplementary material 1. [file 12672_2024_1040_MOESM1_ESM.zip › original images/Migration assay/sw480-12h-siIFMT2.tif]

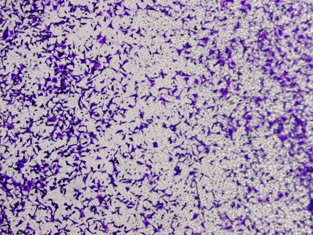

Supplement: Supplementary file 1 — Supplementary material 1. [file 12672_2024_1040_MOESM1_ESM.zip › original images/Migration assay/sw480-12h.tif]

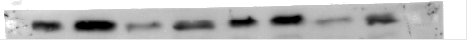

Supplement: Supplementary file 1 — Supplementary material 1. [file 12672_2024_1040_MOESM1_ESM.zip › original images/Western blotting/Fig.1 IFITM2-1.tif]

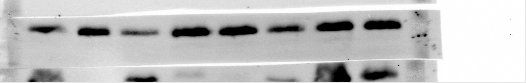

Supplement: Supplementary file 1 — Supplementary material 1. [file 12672_2024_1040_MOESM1_ESM.zip › original images/Western blotting/Fig.1 IFITM2-2.tif]

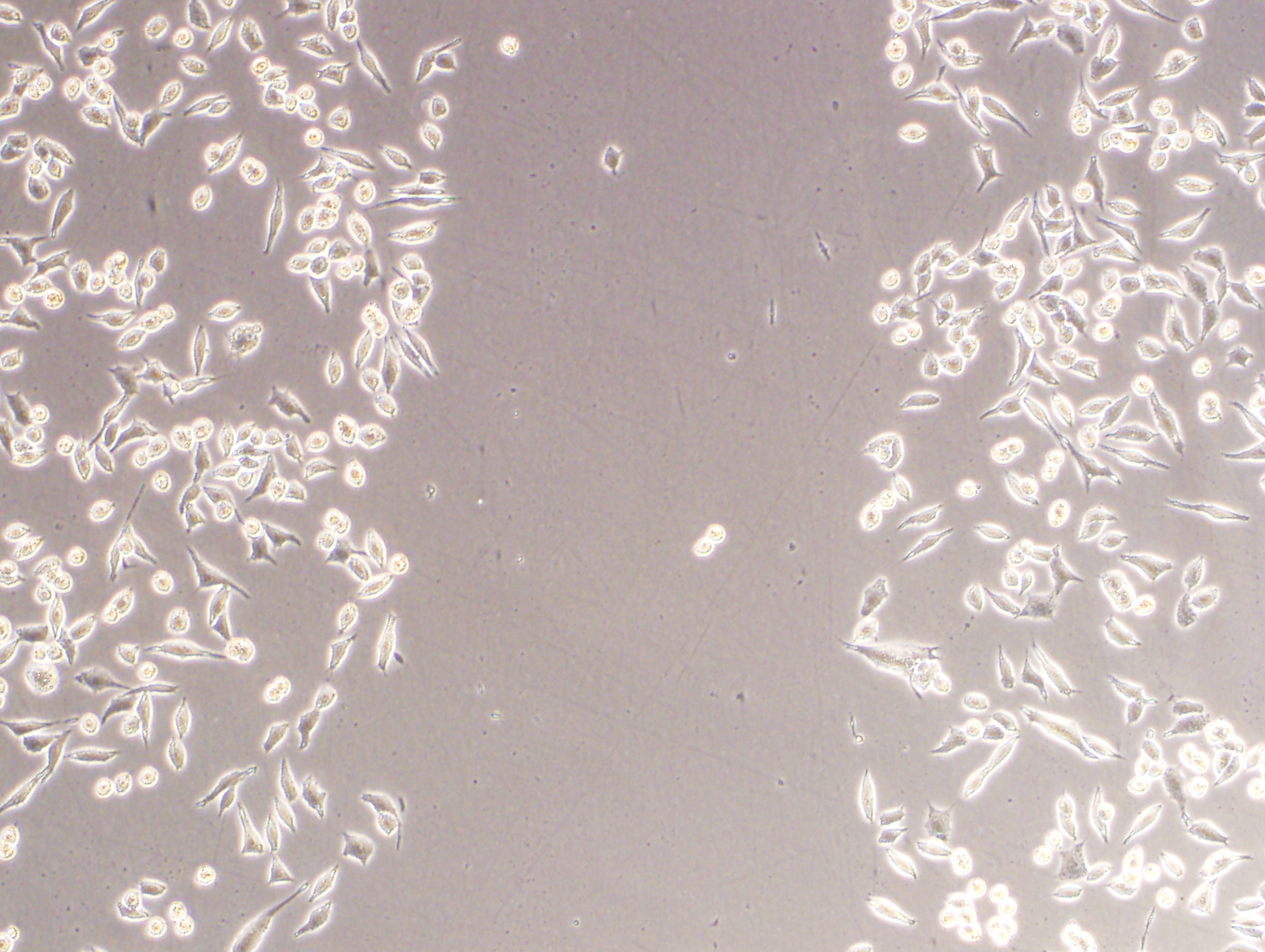

Supplement: Supplementary file 1 — Supplementary material 1. [file 12672_2024_1040_MOESM1_ESM.zip › original images/Wound healing assay/hct116-ctl-0h.tif]

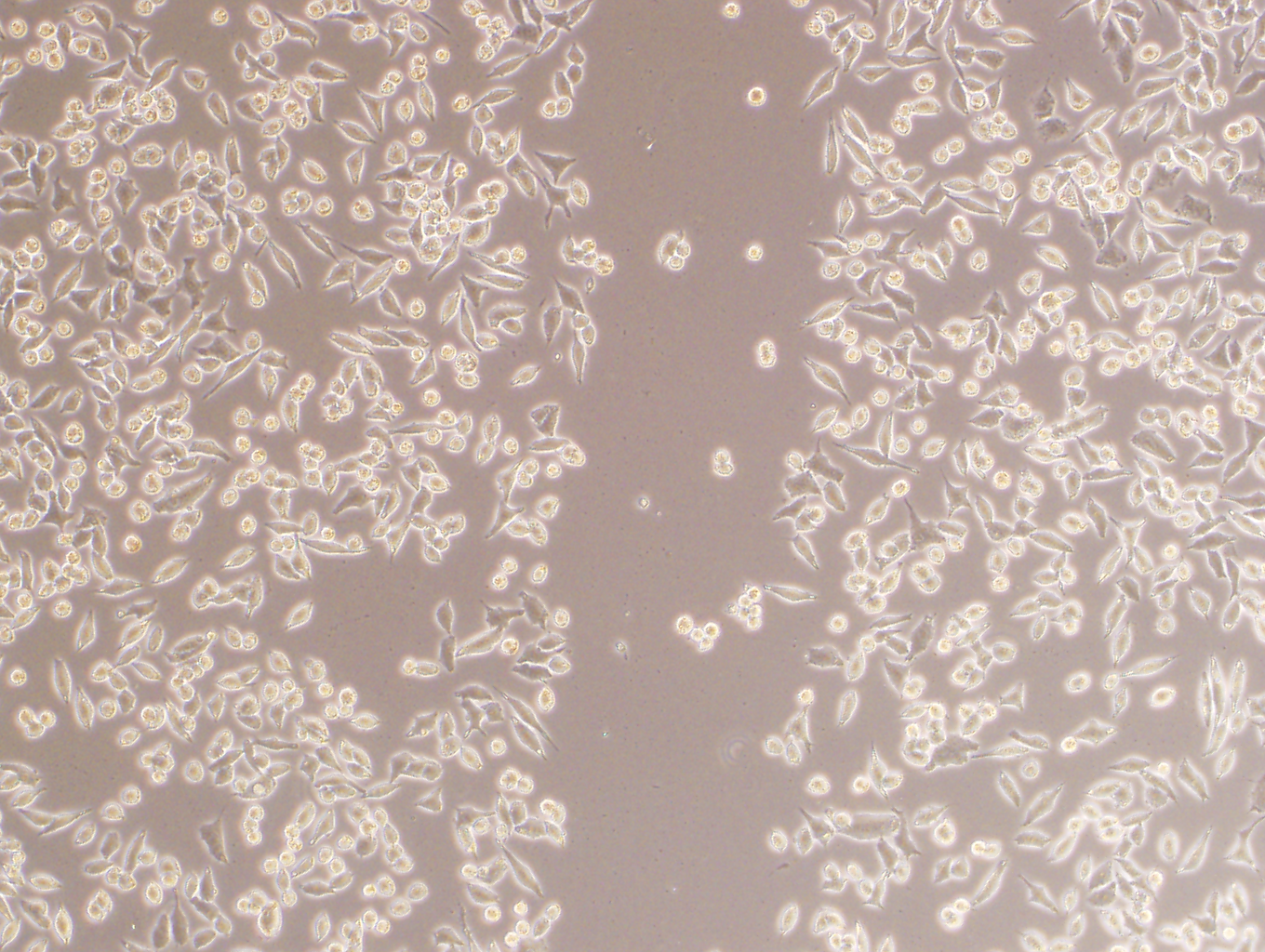

Supplement: Supplementary file 1 — Supplementary material 1. [file 12672_2024_1040_MOESM1_ESM.zip › original images/Wound healing assay/hct116-ctl-12h.tif]

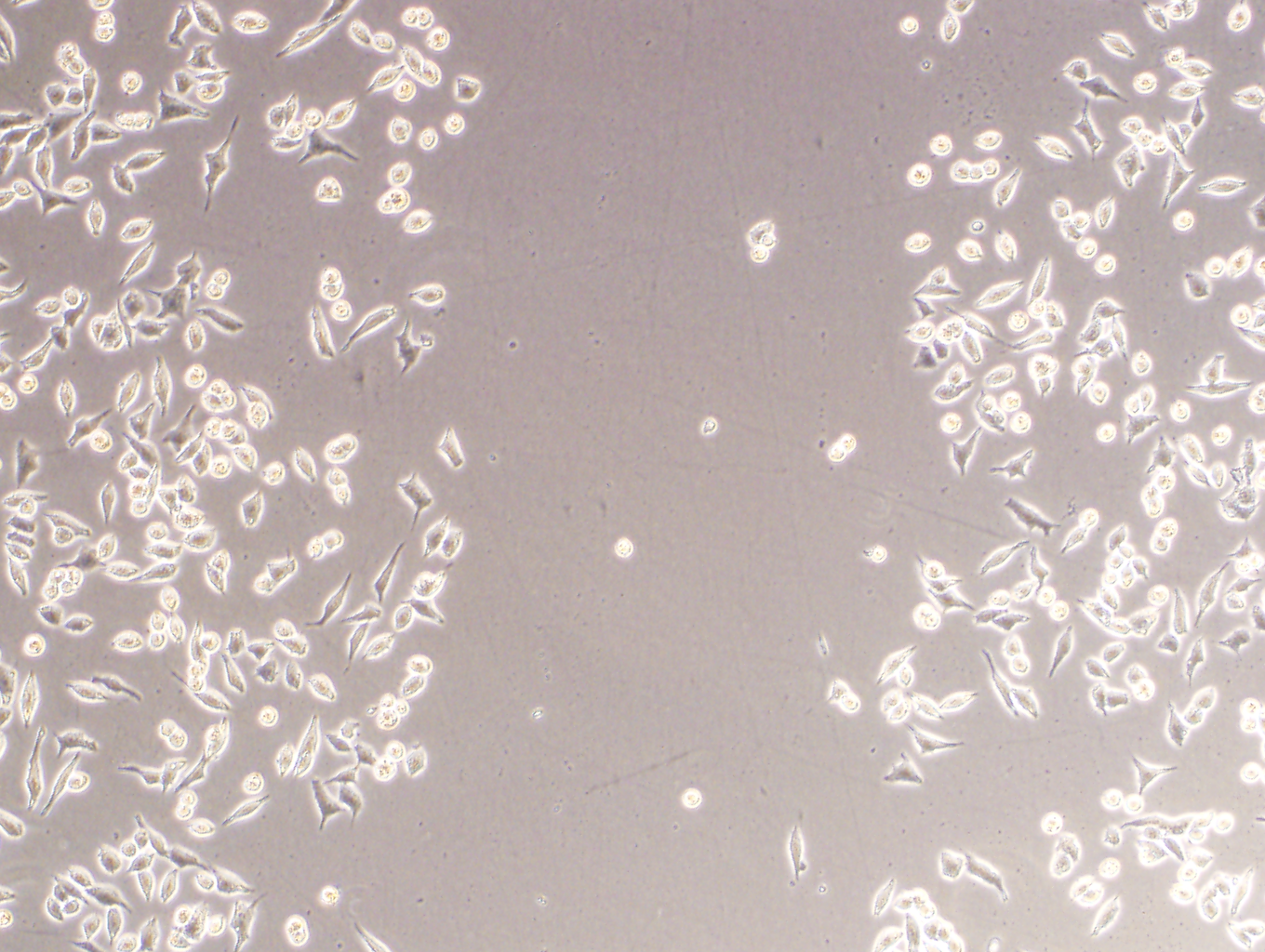

Supplement: Supplementary file 1 — Supplementary material 1. [file 12672_2024_1040_MOESM1_ESM.zip › original images/Wound healing assay/hct116-siIFITM2-0h.tif]

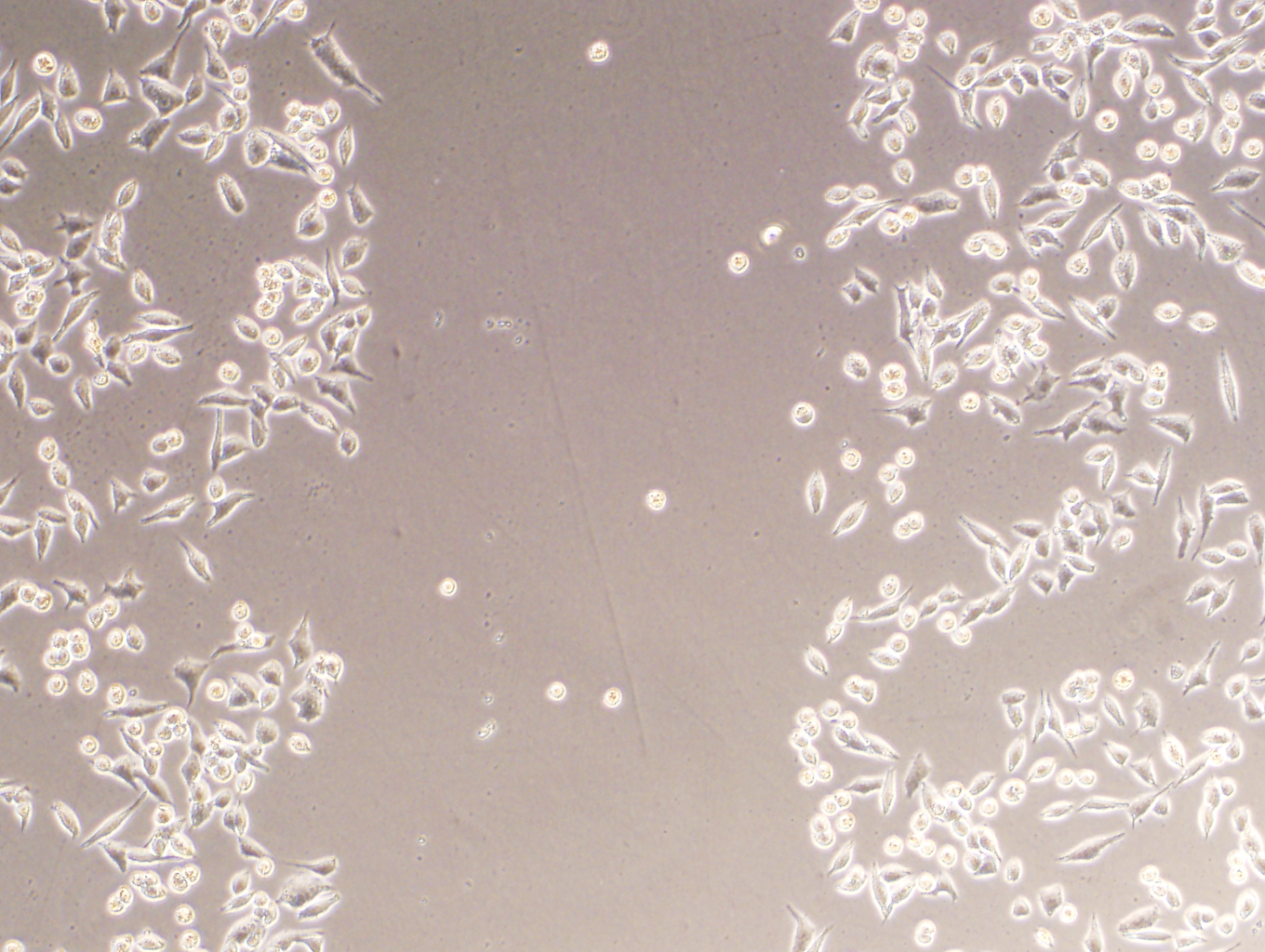

Supplement: Supplementary file 1 — Supplementary material 1. [file 12672_2024_1040_MOESM1_ESM.zip › original images/Wound healing assay/sw480-ctl-0h.tif]

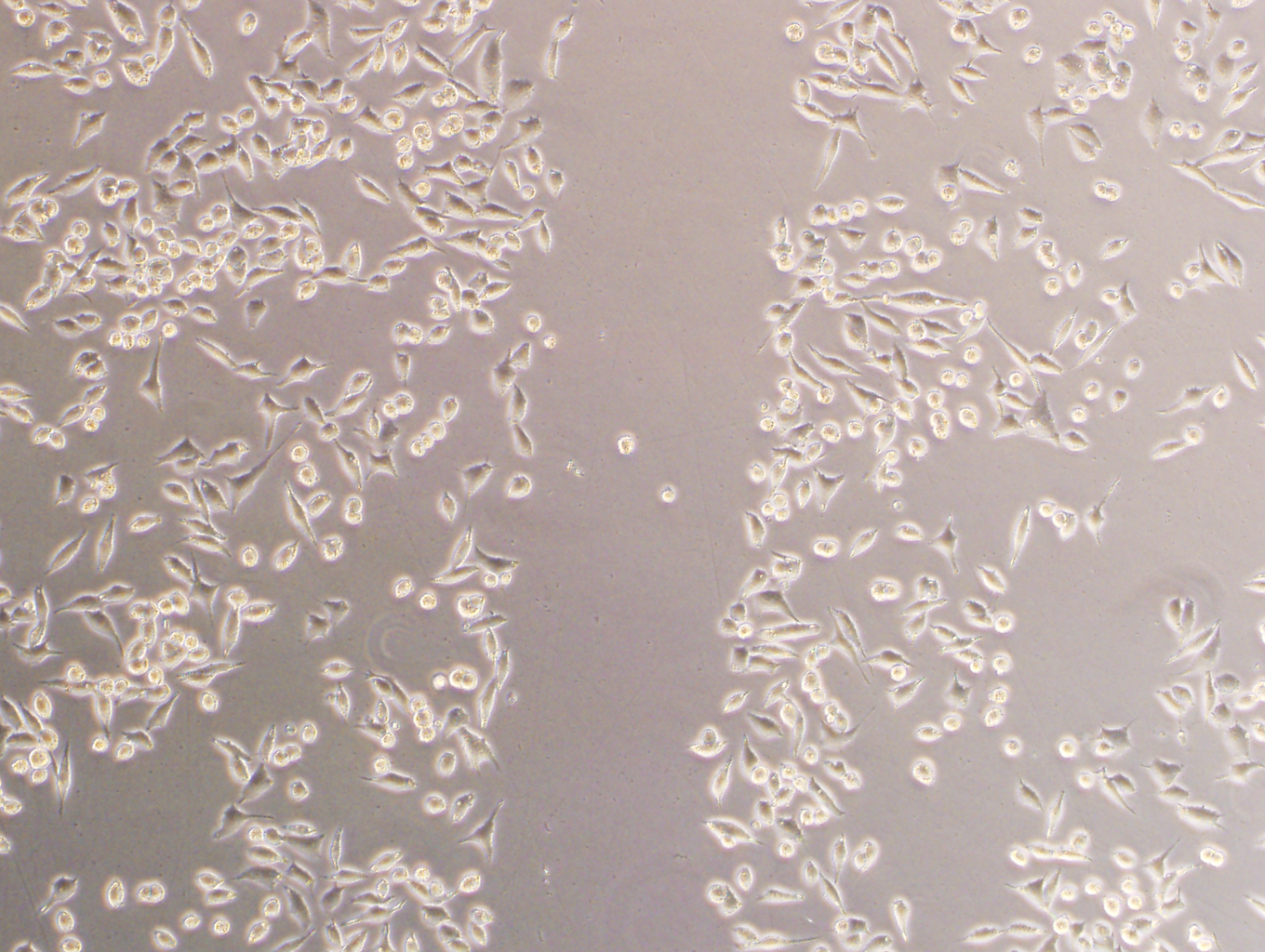

Supplement: Supplementary file 1 — Supplementary material 1. [file 12672_2024_1040_MOESM1_ESM.zip › original images/Wound healing assay/sw480-ctl-12h.tif]

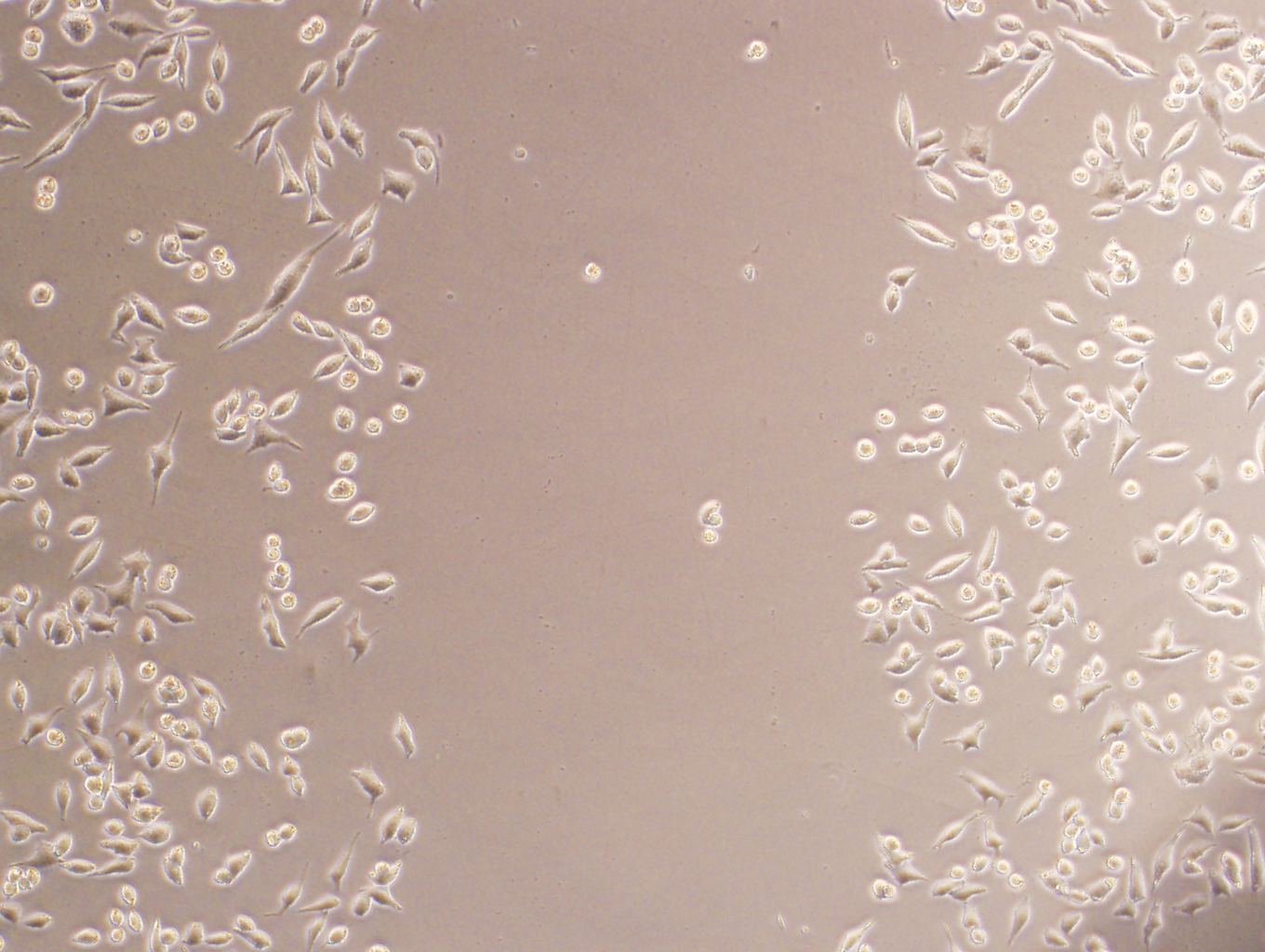

Supplement: Supplementary file 1 — Supplementary material 1. [file 12672_2024_1040_MOESM1_ESM.zip › original images/Wound healing assay/sw480-siIFITM2-0h.tif]

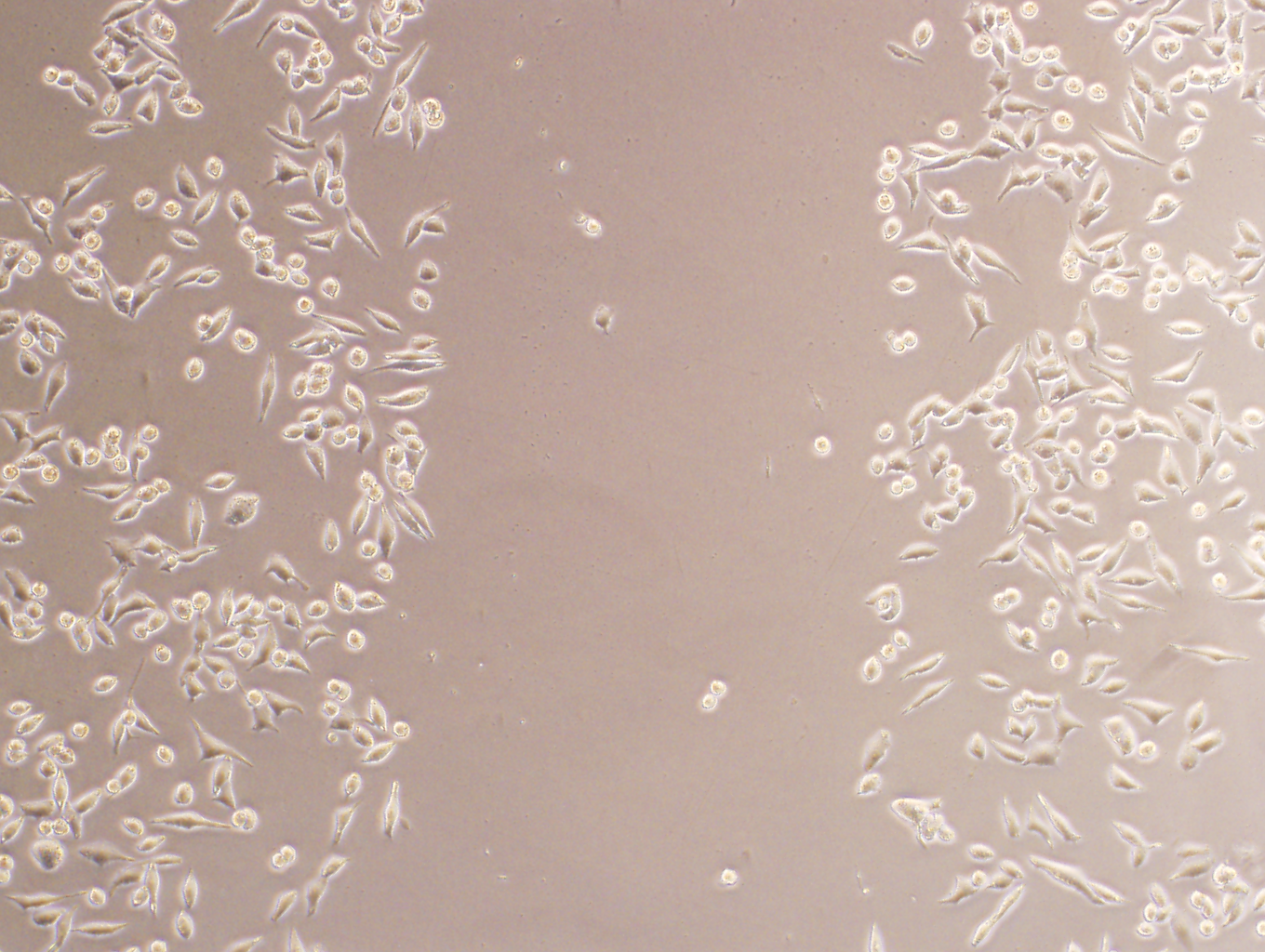

Supplement: Supplementary file 1 — Supplementary material 1. [file 12672_2024_1040_MOESM1_ESM.zip › original images/Wound healing assay/sw480-siIFITM2-12h.tif]
